# Discovery and structural studies of histone demethylases

**DOI:** 10.3389/freae.2025.1594400

**Published:** 2025-05-30

**Authors:** Longfei Peng, Xinze Li, Hao Yang, Haonan Chen, Yue Yang, Shunfeng Peng

**Affiliations:** ^1^ Department of Chemistry, College of Sciences, Shanghai University, Shanghai, China; ^2^ Longevity and Aging Institute, The Shanghai Key Laboratory of Medical Epigenetics, Institutes of Biomedical Sciences, Zhongshan Hospital, Fudan University, Shanghai, China; ^3^ Lab Center, School of Electrical and Information Engineering, Anhui University of Technology, Ma’anshan, Anhui, China

**Keywords:** histone demethylases, lysine demethylation, argine demethylation, structure, KDM family

## Abstract

The discovery and structural elucidation of histone demethylases represent a groundbreaking advancement in the field of epigenetics. Histone methylation, a critical chromatin modification, was long regarded as irreversible until the identification of histone demethylases overturned this paradigm. In 2004, the discovery of the first histone demethylase, LSD1 (Lysine-Specific Demethylase 1), unveiled the dynamic regulatory mechanisms governing methylation modifications. Subsequent identification of the JmjC domain-containing demethylase family further expanded the diversity and functional repertoire of these enzymes. Structural biology studies have revealed the molecular mechanisms by which these enzymes remove methyl groups via oxidation or hydroxylation reactions, providing key insights into their substrate specificity and catalytic processes. This article will provide a concise overview of the discovery history, fundamental structures, and functional mechanisms of histone demethylases, summarize research progress on identified histone demethylases, and offer novel insights and offer novel insights and suggestions for fundamental research on sites where demethylases remain undiscovered.

## Introduction

Post-translational modifications (PTMs) represent a fundamental regulatory mechanism that ubiquitously modulates protein function, localization, and interactions within cellular systems. They alter the properties and functions of proteins by adding or removing specific chemical groups to or from the side chains of amino acids. Extensive research has identified a diverse array of PTMs, including but not limited to phosphorylation, ubiquitination, glycosylation, as well as numerous other residue-specific chemical alterations (Kouzarides, 2007). Among these modifications, protein methylation represents a ubiquitous and reversible PTM that is universally conserved across all cell types, exerting profound regulatory effects on virtually all aspects of cellular processes and biological functions. In contrast to genetic alterations such as mutations, deletions, amplifications, and rearrangements, epigenetic modifications including PTMs represent an energy-efficient regulatory mechanism that enables organisms to modulate phenotypic expression with relatively low metabolic cost.

In 1884, Albrecht Kossel employed chemical extraction methods to isolate an alkaline protein rich in arginine and lysine from avian erythrocytes (which are nucleus-rich). As this protein was primarily localized in the cell nucleus (histos, Greek for “tissue”), Kossel designated it as “histone.” In addition, Kossel demonstrated that these proteins are positively charged under acidic conditions and can bind to negatively charged DNA, laying the foundation for further understanding of DNA-protein interactions. This seminal work earned Kossel the 1910 Nobel Prize in Physiology or Medicine. This important discovery also made people realize that there is not only DNA in the nucleus, but also numerous histones that interact with DNA and affect DNA replication and transcription, promoting the rise of epigenetics. In 1974, after the discovery of the double helix structure of DNA, Roger Kornberg discovered and elucidated the mechanism of action between histones and DNA through X-ray diffraction crystal analysis and biochemical experiments, namely, the nucleosome model (Kornberg, 1974). Roger Kornberg was also awarded the 2006 Nobel Prize in Chemistry for this. The discovery of nucleosome models has promoted the study of histone modifications such as methylation and acetylation, greatly expanding the research scope of epigenetics. In summary, histones constitute fundamental structural components of chromatin, comprising five major subtypes: H1, H2A, H2B, H3, and H4. The nucleosome, recognized as the basic unit of chromatin organization during transcriptional processes, is typically formed by 146 base pairs of DNA wrapped around an octameric histone core consisting of H2A, H2B, H3, and H4. Protein methylation was initially identified as a post-translational modification occurring on histones. Subsequent research has established that histone methylation plays a crucial regulatory role in both transcriptional activation and repression of gene expression. In 1959, Ambler and Rees made the seminal discovery of ε-N-methyl-lysine in prokaryotic organisms (Ambler and Rees, 1959). Subsequently, Murray and Allfrey independently reported in 1964 that the abundance of this modified amino acid in mammalian histones exhibited a strong correlation with transcriptional activity (Murray, 1964; Allfrey et al., 1964). The functional significance of histone methylation was further elucidated in 1999 when site-specific amino acid methylation and N-methyltransferases were experimentally demonstrated to participate in transcriptional activation regulation (Chen et al., 1999; Strahl et al., 1999). A year later, a groundbreaking discovery was made by Jenuwein’s research team, who identified SUV39H1 as the first real histone N-methyltransferase capable of catalyzing H3K9 lysine methylation (Rea et al., 2000). Following the identification of the catalytic SET domain within SUV39H1, a multitude of N-methyltransferases containing analogous domains that catalyze methylation at distinct histone sites have been subsequently characterized. Current research has identified three distinct methylation states for lysine residues: mono-methylation, di-methylation, and tri-methylation (denoted as Kme1, Kme2, and Kme3). Furthermore, arginine residues also exhibit three methylation patterns: mono-methylation, symmetric di-methylation, and asymmetric di-methylation (denoted as Rme1, Rme2s, and Rme2a).

The discovery of histone demethylases presented substantially greater experimental challenges compared to the identification of histone methyltransferases. The substantial bond energy associated with methyl groups and the crucial role of methylation in cellular transcription and development processes led to persistent scientific debates regarding the existence of demethylation enzymes for several decades following the initial discovery of amino acid methylation. In 2004, Professor Yang Shi and Professor Yujiang Geno Shi identified an enzymatic activity capable of removing mono- and di-methyl groups from H3K4 within KIAA0601, a component of the CtBP transcriptional repressor complex (Shi et al., 2004). This protein was subsequently reclassified as LSD1 (Lysine-specific Histone Demethylase 1). This discovery conclusively resolved the long-standing scientific debate regarding the existence of active demethylation processes in mammalian systems. In 2005, Yi Zhang’s research team made another significant breakthrough with the identification of JHDM1A (JmjC domain-containing Histone Demethylase 1A), a distinct class of lysine demethylase (Tsukada et al., 2006). Unlike LSD1, JHDM1A contains a JmjC domain and requires Fe^2+^ ions and α-ketoglutarate (α-KG) as essential cofactors for its demethylation activity. The subsequent discovery of LSD2, an LSD1 homolog, along with the identification of additional JmjC domain-containing enzymes with histone demethylase activity, has progressively established a comprehensive lysine demethylase system. Notably, following the discovery of JHDM1 as the first JmjC domain-containing protein, researchers have systematically characterized a total of 30 proteins harboring this conserved domain (Klose et al., 2006) (Figure 1). These proteins have been classified into seven different families based on phylogenetic analysis of their JmjC domain sequences and the presence of additional functional domains. Despite these significant advances, the enzymatic machinery responsible for demethylation at several histone methylation sites, including lysine H3K79me1/me2/me3, arginine H3R2, H4R3 and so on, remains elusive.

**FIGURE 1:**
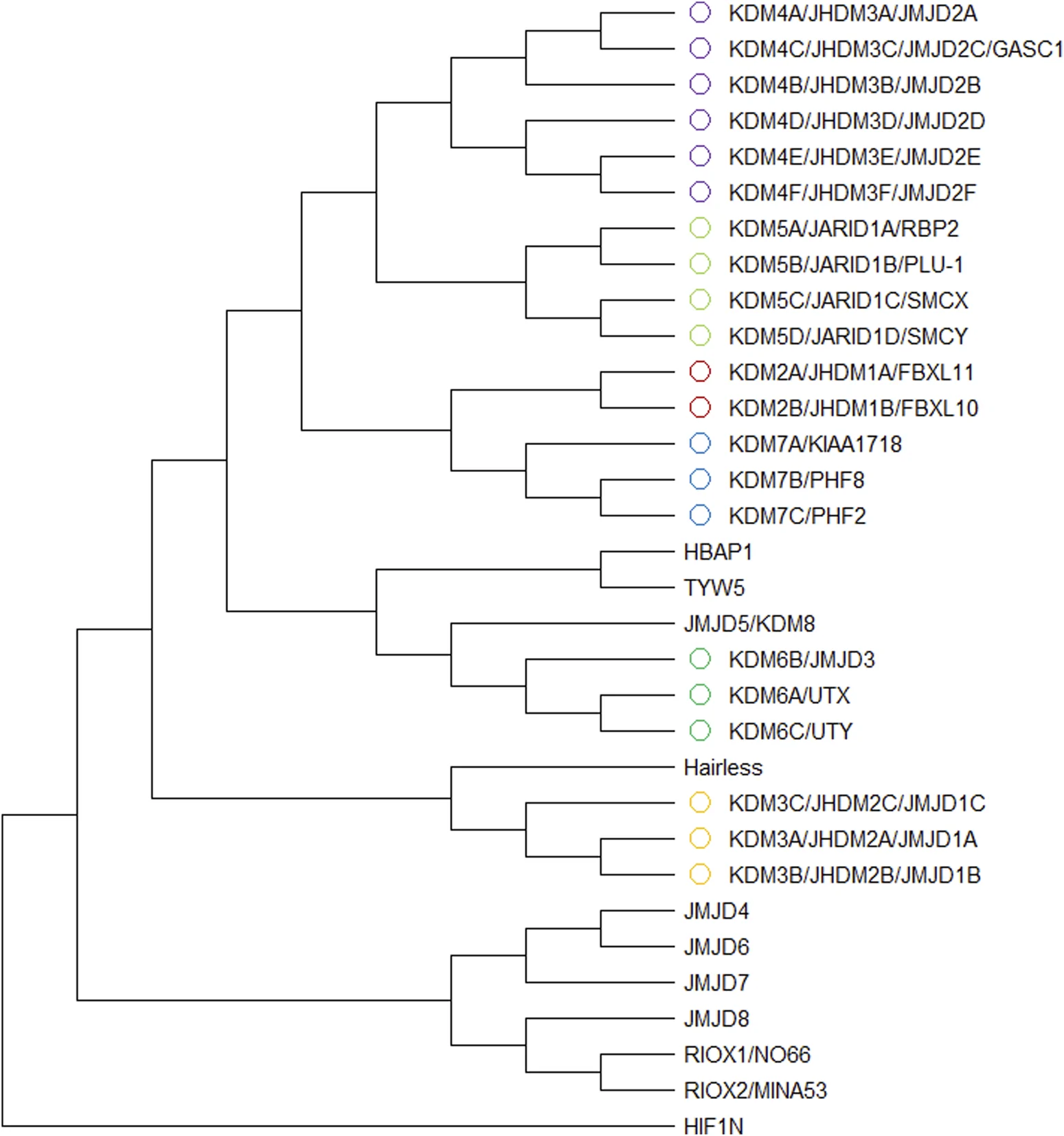
Phylogenetic tree from 32 JmjC family proteins. The whole sequences of them were aligned with the ClustalW algorithm and a phylogenetic tree generated with Mega11. Different colors represent different KDM families.

Histone methylation plays a pivotal role in regulating diverse cellular processes, particularly in modulating transcriptional activity. Therefore, extensive research efforts have been devoted to elucidating the structural architecture, functional mechanisms, and biological significance of histone demethylases. The rapid advancement of structural biology techniques, including X-ray crystallography and cryo-electron microscopy, has provided excellent methods into the three-dimensional structures of these enzymes and their substrate recognition specificity. This review presents a chronological overview of demethylase discovery, with particular emphasis on structural analyses of distinct demethylase families and their functional roles in gene expression regulation. Finally, we summarize current research progress regarding histone methylation sites for which corresponding demethylases remain to be identified.

## Classification of histone demethylases

Histone demethylases are systematically classified into two different categories based on their catalytic mechanisms. The first category comprises flavin adenine dinucleotide (FAD)-dependent amine oxidases, exemplified by LSD1, which catalyzes demethylation through an oxidative reaction mechanism mediated by FAD. The second category encompasses Jumonji C (JmjC) domain-containing dioxygenases that require Fe^2+^ and α-KG as essential cofactors for their catalytic activity. The mechanistic constraint of FAD-dependent amine oxidases necessitates the presence of a free electron pair on the ε-amino group’s nitrogen atom to facilitate the formation of an imine intermediate, which is hydrolyzed to release formaldehyde later. This specific requirement restricts their catalytic activity to mono- and di-methylated lysine residues, rendering them incapable of processing tri-methylated substrates (Figure 2A). And JmjC domain-containing dioxygenases catalyze demethylation through a hydroxylation-oxidation-decarbonylation cascade reaction, utilizing α-KG, Fe^2+^, and molecular oxygen as essential cofactors. This unique catalytic mechanism enables these enzymes to sequentially remove methyl groups from tri-methylated lysine residues, thereby distinguishing them from FAD-dependent amine oxidases in terms of substrate specificity and catalytic capability. It is noteworthy that this enzymatic process may proceed through two different catalytic mechanisms: one requiring conformational rearrangement of α-KG and the other involving spatial flipping of the Fe^2+^ ion within the active site (Chaturvedi et al., 2020) (Figures 2A,B). These alternative mechanistic pathways highlight the structural flexibility and catalytic complexity of JmjC domain-containing dioxygenases in facilitating the demethylation process.

**FIGURE 2:**
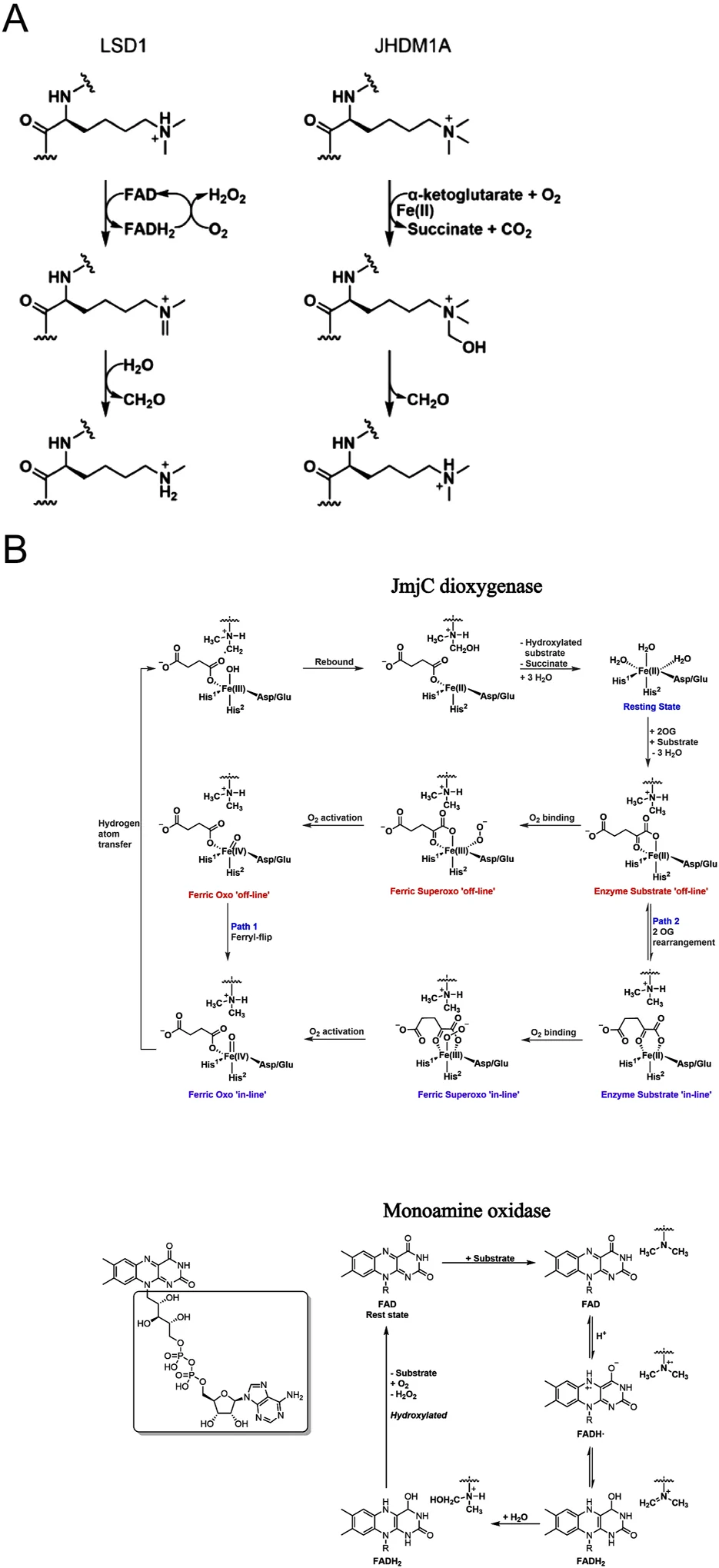
**(A)** The demethylation mechanisms of two enzymes LSD1 and JHDM1A Monoamine oxidase mechanism and dioxygenase mechanism; **(B)** The pathway of monoamine oxidase and two pathways of demethylation by JmjC dioxygenase Fe^2+^ forms an “offline” iron-based oxygen based intermediate (Pathway 1) with O_2_, and the iron-based structure flips, or the binding mode of 2OG C-1 carboxylate salt is changed from “offline” to “online” before O_2_ binding (Pathway 2).

Following the identification of various histone demethylases, the scientific community established a systematic nomenclature to facilitate standardized research communication. In this unified classification system, LSD1 and its sole homolog LSD2 were designated as KDM1A and KDM1B (Allis et al., 2007). And the JmjC domain-containing proteins with confirmed demethylase activity were categorized into the KDM2 through KDM7 families, based on their structural characteristics and functional properties (Allis et al., 2007) (Figure 3).

**FIGURE 3:**
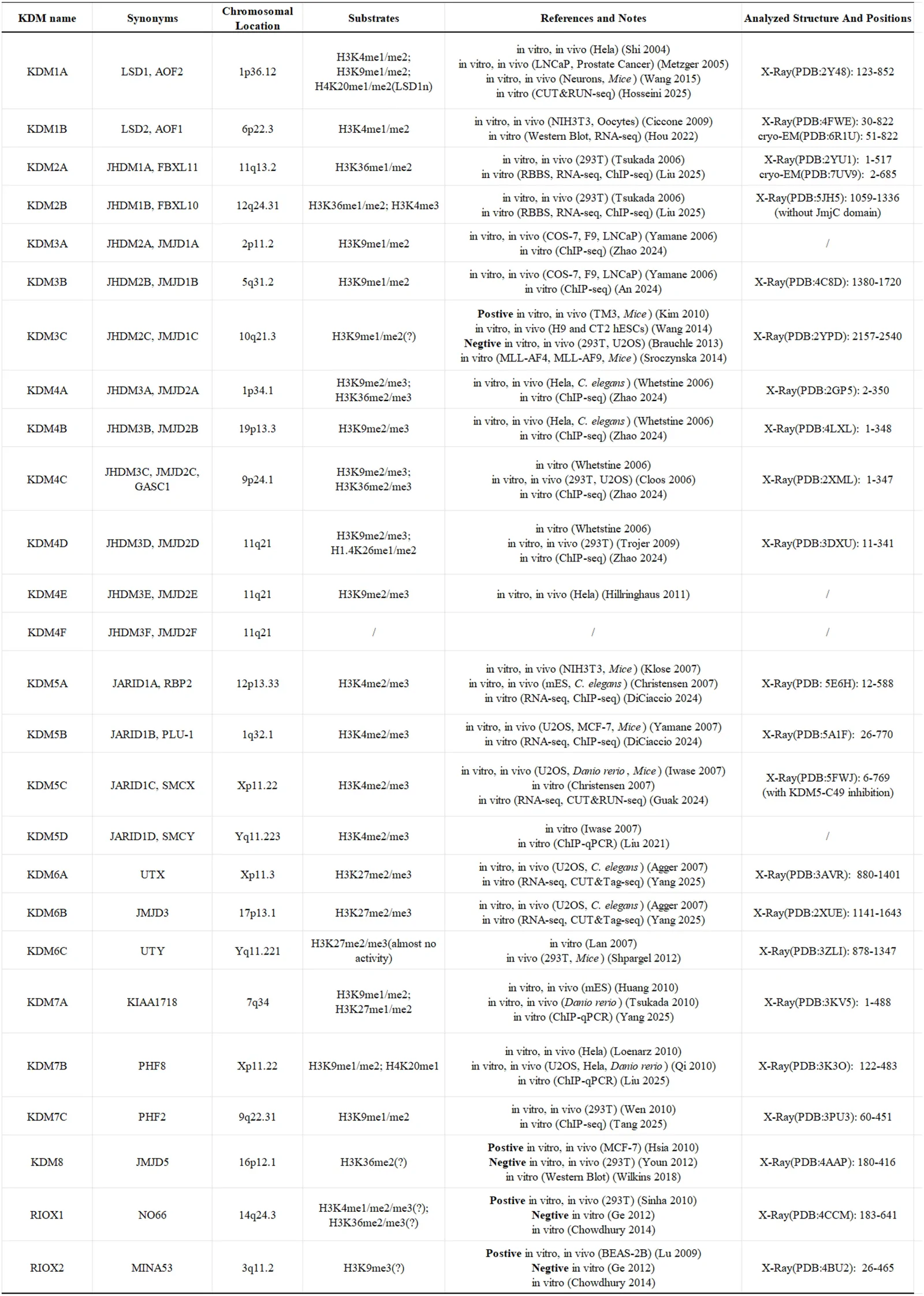
The fundamental details of KDMs family. The question mark (?) indicates a doubt about the substrate of the demethylase (Hosseini et al., 2025; Hou et al., 2022; Liu Y. et al., 2025; Zhao Z. et al., 2024; An et al., 2024; Zhao X. et al., 2024; DiCiaccio et al., 2024; Guak et al., 2024; Liu and Gao, 2021; Yang S-G. et al., 2025; Yang Z-J. et al., 2025; Liu Z. et al., 2025; Tang et al., 2025).

## Discovery of histone demethylases

### KDM1 family

LSD1/KDM1A is the first identified histone demethylase. During their investigation of the CtBP transcriptional repressor complex, Professor Yang Shi and Professor Yujiang Gono Shi discovered that the protein KIAA0601, alternatively known as NPAO/BHC110, possessed histone demethylation activity. Bioinformatics analysis of the protein sequence revealed the presence of a potential amine oxidase domain. Following extensive but unsuccessful efforts to identify its physiological substrate, the researchers hypothesized that the substrate might be structural analogs of polyamines, specifically methylated basic amino acids such as lysine or arginine residues. This insightful hypothesis ultimately led to the discovery of the protein’s demethylase activity specifically targeting histone H3K4. KDM1A consists of an N-terminal SWIRM (Swi3p/Rsc8p/Moira) domain, a central TOWER domain, and a C-terminal amine oxidase-like (AOL) domain that is flanked by the TOWER domain (Shi et al., 2004; Lee et al., 2005) (Figure 4A). The AOL domain constitutes the catalytic core responsible for the demethylation reaction. Although separated in sequence by the TOWER domain, the two AOL segments spatially assemble to form a functional amine oxidase unit (Yang et al., 2006). The SWIRM domain mediates nucleosome targeting, enabling KDM1A to precisely localize to specific chromatin sites (Da et al., 2006). The TOWER domain, extending from the core region as a tower-like structure, facilitates interaction with the CoREST transcriptional corepressor complex (Lee et al., 2005) (Figure 4B). In addition to CoREST, KDM1A forms integral components of various protein complexes, including BHC80, HDAC1/2, and BRAF35 (Hakimi et al., 2002; Shi et al., 2005). Initial studies revealed that KDM1A primarily demethylates H3K4me1/me2 on peptide substrates and histones (Lee et al., 2005). However, upon complex formation with CoREST, the KDM1A-CoREST complex acquires the capacity to recognize nucleosomes as its physiological substrate (Shi et al., 2005). This phenomenon underscores the crucial role of the TOWER domain in mediating KDM1A’s demethylation function *in vivo*. Interestingly, when KDM1A forms a ligand-dependent complex with the activated estrogen receptor (ER), its substrate specificity undergoes a remarkable shift from H3K4me1/me2 to H3K9me1/me2 (Metzger et al., 2005). This complex cooperates with JMJD2C/KDM4C to remove repressive H3K9me1/me2/me3 marks, thereby maximally activating ER target gene expression through coordinated epigenetic regulation (Wissmann et al., 2007). Furthermore, the neuron-specific isoform LSD1n, generated through alternative splicing of KDM1A, acquires unique demethylase activity towards H4K20me1/me2 (Wang et al., 2015). LSD1n regulates memory and learning processes by occupying enhancers, promoters, and transcribed regions of neuronal development-related genes, thereby modulating transcriptional initiation and elongation during neurogenesis (Wang et al., 2015). And another alternative splicing isoform KDM1A+8a can interact with protein supervillain to mediate the demethylation of H3K9me2 and promote neuronal differentiation (Laurent et al., 2015). These examples illustrate that some special demethylases can undergo significant alterations in their catalytic configurations, substrate specificity, and biological functions through either association with different protein partners or alternative splicing events. Besides histone H3, KDM1A also targets non-histone proteins as substrates, including DNMT1, p53, STAT3, and β-catenin (Wang et al., 2009; Huang et al., 2007; Yang J. et al., 2010; Mouradian et al., 2024). During early embryonic stem cell development, KDM1A modulates global DNA methylation patterns and embryonic development by regulating DNMT1 methylation status without compromising its catalytic activity (Wang et al., 2009). The tumor suppressor and transcriptional activator p53 is similarly regulated by KDM1A, which suppresses p53-mediated transcriptional activation and apoptotic function through demethylation of p53-K370me1/me2 (Huang et al., 2007). KDM1A also regulates diverse biological processes, including immune system modulation (Qiu et al., 2024; Pallavicini et al., 2024), aging retardation (Del Blanco et al., 2024), and stem cell differentiation/proliferation, through its histone and non-histone demethylase activity. This regulation is achieved by controlling transcriptional levels of various genes, such as PD-L1 (Qiu et al., 2024; Pallavicini et al., 2024), aging related genes silenced by PRC2 (also influenced by topological structure of KDM1A) (Del Blanco et al., 2024), β-catenin (Mouradian et al., 2024), thereby influencing the progression of these critical cellular activities. At last, LSD1 overexpression has been observed to drive tumor cell proliferation and metastasis across various cancer cell lines (Huang et al., 2025; Wang et al., 2025; Jing et al., 2024; Yang et al., 2024; Qu et al., 2024). For example, in hepatocellular carcinoma, KDM1A promotes tumor progression through its H3K4me1/me2 demethylase activity by suppressing transposable elements near the HNF4A gene and silencing HNF4A expression (Jing et al., 2024). These researches collectively establish KDM1A as a promising therapeutic target with significant potential for cancer treatment, particularly in combination therapies with existing pharmacological agents.

**FIGURE 4:**
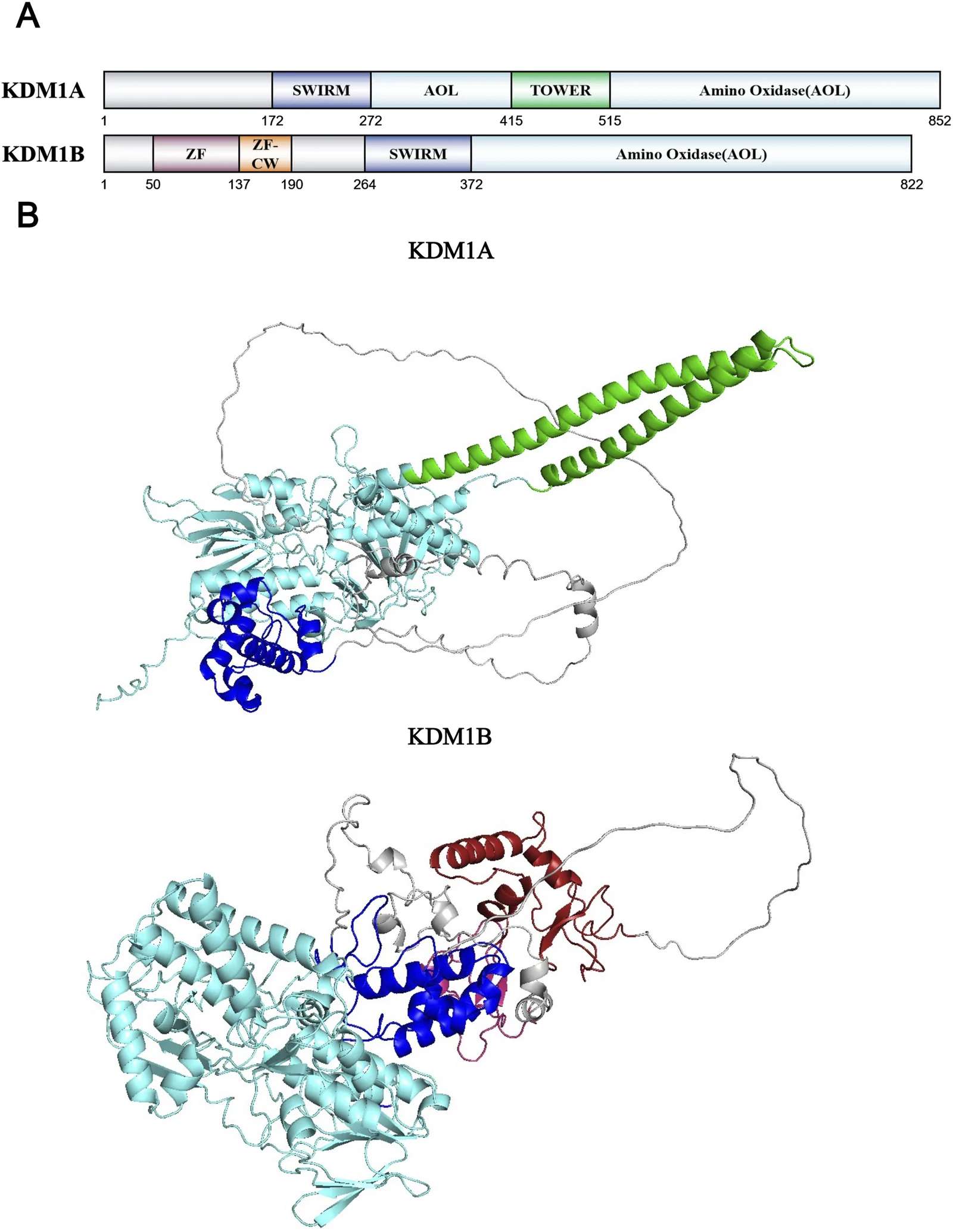
**(A)** Domains and sequences of KDM1 Family. The sequences of amino acids are annotated from UniProt database (KDM1A: O60341; KDM1B: Q8NB78); **(B)** Structure of KDM1 family proteins from AlphaFold. The color of each structural domain corresponds to **(A)**. Above proteins’ AOL domains have been analyzed by X-ray (PDB ID: KDM1A-2Y48; KDM1B-4FWE) and cryo-EM (PDB ID: KDM2B-6R1U).

KDM1B, also known as LSD2 or AOF1, represents the homolog of KDM1A. Identified in 2009, KDM1B shares structural features with KDM1A, containing both amine oxidase and SWIRM domains, and requires FAD as a cofactor (Ciccone et al., 2009). KDM1B specifically catalyzes the removal of mono- and di-methyl groups from H3K4, demonstrating substrate specificity similar to KDM1A (Ciccone et al., 2009; Fang et al., 2010). Unlike KDM1A, KDM1B lacks the TOWER domain, which consequently prevents its interaction with the CoREST corepressor complex. However, KDM1B possesses a unique N-terminal domain featuring a new C4H2C2-type zinc finger domain and a special CW-type zinc finger domain, which can recognize and bind histone H3K4me3 specially and inhibit transcription by binding to DNA in a demethylase-independent mechanism (Perry and Zhao, 2003; Yang Z. et al., 2010; He et al., 2010; Hoppmann et al., 2011) (Figure 4A). Crystallographic analysis shows that KDM1B and KDM1A share similar core catalytic structures. KDM1B also requires the coordinated function of all three domains for proper substrate assembly, which proves the significant role of zinc finger domains in KDM1B’s catalytic mechanism (Zhang et al., 2013) (Figure 4B). Obviously, KDM1B exhibits different cofactor requirements and binding partners compared to KDM1A. Currently, NPAC/GLYR1 has been identified as the sole binding partner involved in KDM1B’s demethylation process (Fang et al., 2013). This protein facilitates H3K4 demethylation by forming a novel hydrophobic pocket with KDM1B to accommodate the H3L20 side chain, thereby stabilizing the KDM1B-H3 interaction (Fang et al., 2013). Furthermore, KDM1B can form active complexes with histone methyltransferases G9a, NSD3 and some transcription elongation factors to modulate histone methylation in gene coding regions and promote RNA polymerase II-mediated transcriptional elongation (Fang et al., 2010). Although non-histone substrates of KDM1B remain to be identified, KDM1B also plays significant roles in regulating gene transcription and other biological processes (Fang et al., 2010; van Essen et al., 2010). Recent studies have revealed that IFN-I can reprogram cancer cells into a more aggressive stem-like phenotype and drives immune evasion through upregulation of KDM1B (Musella et al., 2022). In summary, while the KDM1 family has been extensively studied, research on KDM1B is relatively scarce. Therefore, KDM1B presents great unexplored potential in terms of its biological functions and mechanisms in human physiological processes and disease pathogenesis.

### KDM2 family

One year following the discovery of LSD1/KDM1A, the first members of the JmjC dioxygenase family, KDM2A (also known as JHDM1A or FBXL11) and KDM2B (also known as JHDM1B or FBXL10), were successfully identified (Tsukada et al., 2006). Professor Yi Zhang’s team drew upon previous research on DNA demethylation, which showed that 1-methyladenine and 3-methylcytosine modifications could be oxidatively removed by AlkB family proteins through an α-KG and Fe^2+^-dependent mechanism (Falnes et al., 2002; Trewick et al., 2002). Because of the structural similarity between methylated lysine and these methylated bases, they hypothesized the existence of another amino acid demethylation process resembling the DNA demethylation mechanism. Based on this hypothesis, they identified an unannotated F-box protein, FBXL11 (Winston et al., 1999; Cenciarelli et al., 1999). Besides the F-box domain, this protein contains a JmjC domain, a CXXC zinc finger domain, a PHD domain, and multiple leucine-rich repeat (LRR) domains (Figure 5A). By constructing truncated proteins with a single domain deletion, they confirmed that the JmjC domain of FBXL11 is indispensable for the demethylation reaction. This finding also aligns with previously proposed functions and structural features of the JmjC domain: it exhibits high sequence similarity to cupin superfamily members, which represent a class of metalloenzymes utilizing zinc ions as cofactors across archaea, bacteria, and eukaryotes (Balciunas and Ronne, 2000; Dunwell et al., 2000; Clissold and Ponting, 2001). Then, they used isotopic labeling techniques to demonstrate that both FBXL11 and its homolog FBXL10 possess demethylase activity towards H3K36me1/me2, and renamed FBXL11 as JHDM1A and FBXL10 as JHDM1B (Tsukada et al., 2006). X-ray crystallographic analysis revealed that the specificity of KDM2A towards H3K36me1/me2 comes from its “Molecular threading mechanism” (Cheng et al., 2014) (Figure 5B). Firstly, the JmjC domain sequences of all JmjC families are highly conserved, especially His, Asp/Glu (acidic amino acid with carbonyl groups), which form coordination bonds with Fe^2+^ ions, and Lys, Asn, Thr/Tyr (with polar group such as carboxyl groups), which form hydrogen bonds and intermolecular forces with α-KG (Klose et al., 2006; Chen et al., 2006). After Fe^2+^ ion forms coordination bonds with the carbonyl group of α-KG and the oxygen atom of water, these components collectively establish a stable octahedral coordination geometry with the JmjC domain, which serves as the chemical and structural foundation for the enzymatic reaction (Chen et al., 2006). Following initial anchoring of H3K36me1/me2 to KDM2A’s JmjC catalytic center, the K323-F324 segment undergoes a conformational change, forming a ring-like structure with existing segments. This structure only allows the smallest amino acid glycine to pass through, corresponding to Gly33 and Gly34 preceding H3K36. After the H3 peptide chain passes through, the peptide bond between Gly33 and Gly34 forms a hydrogen bond with the cyclic structure to further stabilize the conformation. In addition to these core amino acids, other H3 residues, including Pro38 and Tyr41, contribute to KDM2A’s substrate specificity towards H3K36 through complementary interactions. The catalytic center of KDM2A is also relatively small, measuring 246.96 Å (KDM4A with substrate H3K36me3 is 722.36 Å) (Yu et al., 2010). Unlike KDM2A, KDM2B also can specifically remove the methyl group on H3K4me3 to inhibit ribosomal RNA transcription, but it has no activity on H3K4me2 (Frescas et al., 2007). In addition, there is still limited research on the F-box domain of the KMD2 family. Only the F-box domain of KDM2B is known to bind to CUL1-RING ubiquitin ligase complexes (Han et al., 2016). The KDM2 family also has a truncated subtype KDM2A/B-SF without the JmjC domain. Due to its lack of demethylation activity, this is only an introduction without further discussion.

**FIGURE 5:**
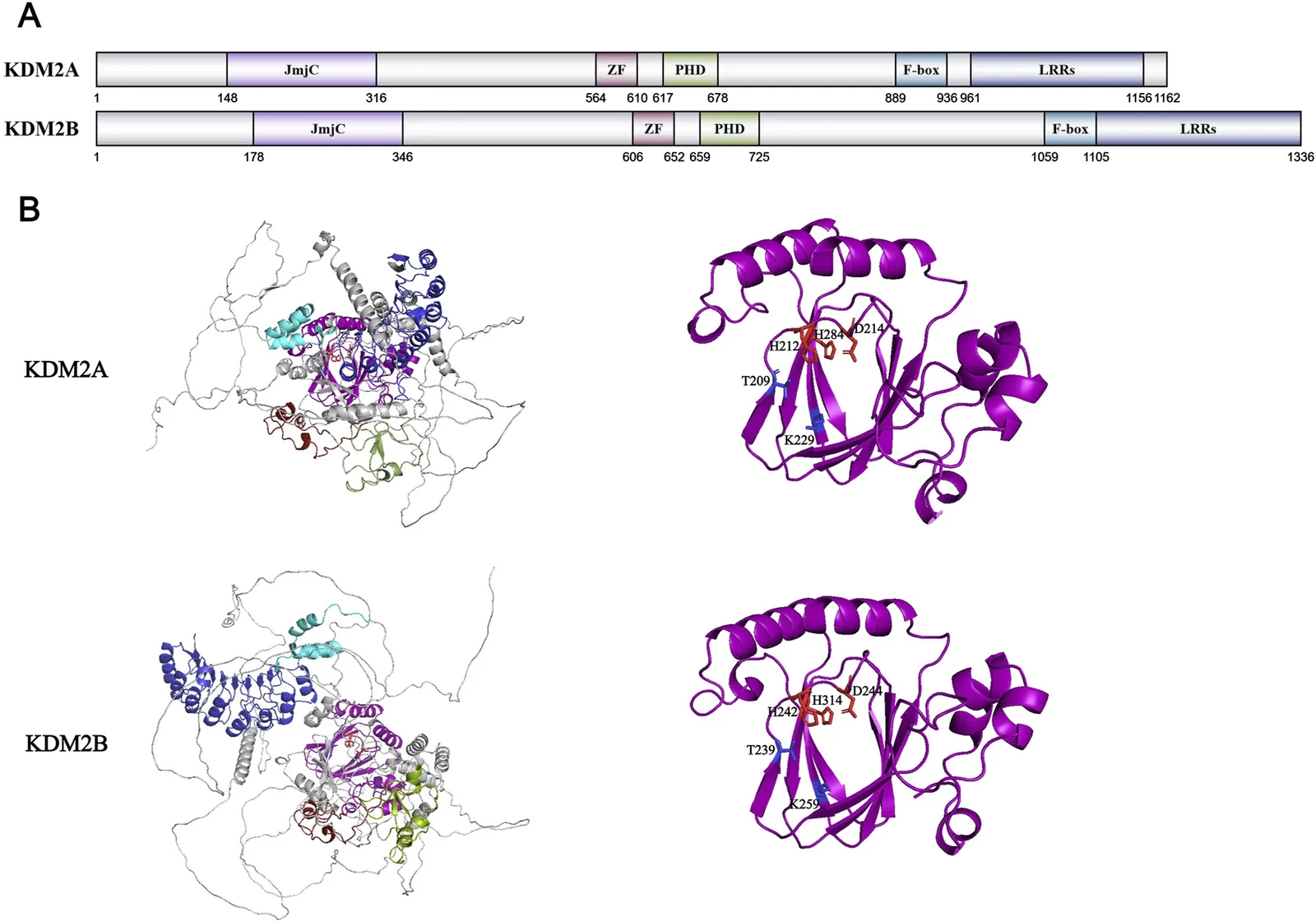
**(A)** Domains and sequences of KDM2 Family. The sequences of amino acids are annotated from UniProt database (KDM2A: Q9Y2K7; KDM2B: Q8NHM5); **(B)** Structure of KDM2 family proteins from AlphaFold. The color of each structural domain corresponds to **(A)**. The amino acid required for Fe^2+^/α-KG binding sites are shown in red/blue respectively in stick form. The core pocket of KDM2A has been analyzed by X-ray (PDB ID: KDM2A-2YU1) and cryo-EM (PDB ID: KDM2A-7UV9). KDM2B has no structural analysis on core pocket. The core pocket structure analysis of KDM2B is based on AlphaFold simulation data and KMD2A data.

The KDM2 family also recognizes non-histone proteins as substrates. KDM2A demethylates RelA (p65) at K218 and K221 within the NF-κB signaling pathway, thereby suppressing NF-κB activation in mouse fibroblast cells (Lu et al., 2010). The key protein nuclear β-catenin in the Wnt signaling pathway is demethylated and ubiquitinated by KDM2A and KMD2B, ultimately leading to inhibition of the Wnt signaling pathway (Lu et al., 2015). The KDM2 family (main KDM2B) interacts with Polycomb Repressive Complex 1 (PRC1) to suppress differentiation-associated genes and promote the reprogramming of stemness genes such as Oct4, thereby maintaining stem cell pluripotency and facilitating the regeneration of pluripotent stem cells (Wang et al., 2011; Wu et al., 2013; Zhou et al., 2017; Wang et al., 2018). The KDM2 family also modulates brain region development and neural system repair through its demethylase-dependent regulation of Wnt and MAPK signaling pathways (Zhang B. et al., 2023; Ren et al., 2024; Cao et al., 2025). And during the development of tumors, the KMD2 family exhibits heterogeneity. While KDM2A promotes bladder cancer progression by suppressing RARRES3 expression through H3K36me1/me2 demethylation at its promoter (Lu et al., 2022), it conversely inhibits breast cancer cell proliferation and metastasis by similarly suppressing EGF transcription and its downstream EGF-TSPAN8 pathway (Zhang et al., 2022). Similarly, KDM2B promotes hepatocellular carcinoma progression by modulating IL-6 expression levels (Wei et al., 2025), while in lung and pancreatic cancers, it suppresses tumor development through a demethylase-independent manner that enhances TGF-β activity (Wanna-Udom et al., 2021). The relevant research on the KDM2 family is relatively complete, which also provides a good foundation for the research on other KDM2A abnormal diseases and corresponding targeted drugs.

### KDM3 family

One year after identifying the KDM2 family, Professor Yi Zhang’s team employed similar methodology to characterize the KDM3 family, which are KDM3A-C, also known as JHDM2A-C or JMJD1A-C. Like KDM2, KDM3 and subsequent histone demethylase families all contain JmjC domains and belong to the Fe^2+^- and α-KG-dependent dioxygenase family. In addition to the JmjC domain, the KDM3 family also features a set of C2HC4 zinc finger domains and a special LxxLL leucine rich sequence (Figure 6A). Both the JmjC domain and zinc finger domains are indispensable for the catalytic function of the KDM3 family (Yamane et al., 2006). After demethylation testing of different methylation sites on histones, the specific demethylation site of KDM3A was confirmed to be H3K9me1/me2. In subsequent studies, KDM3B, which shares 59.64% similarity in amino acid sequence with KDM3A, also exhibits H3K9me1/me2 demethylation activity (Brauchle et al., 2013). However, although the important residues involved in cofactor binding of KDM3C/JMJD1C are completely conserved like KDM3A/3B, its activity has shown inconsistent results in different studies. Some experiments showed that KDM3C exhibited H3K9me1/me2 demethylation activity *in vitro* and *in vivo* (Kim et al., 2010; Wang et al., 2014), while others did not show any activity (Brauchle et al., 2013; Sroczynska et al., 2014). It is speculated that the non-conservation of Thr667 in the zinc finger domain, which is crucial for recognizing H3K9me1/me2 in KDM3A/3B, is the reason for the controversy surrounding the activity of KDM3C (Brauchle et al., 2013). The KDM3 family currently does not have a whole structural model constructed through structural biology methods, only a structural model predicted through AlphaFold (Figure 6B). Therefore, there are still no more details to the spatial structure of the KDM3 family and the recognition mechanism for H3K9.

**FIGURE 6:**
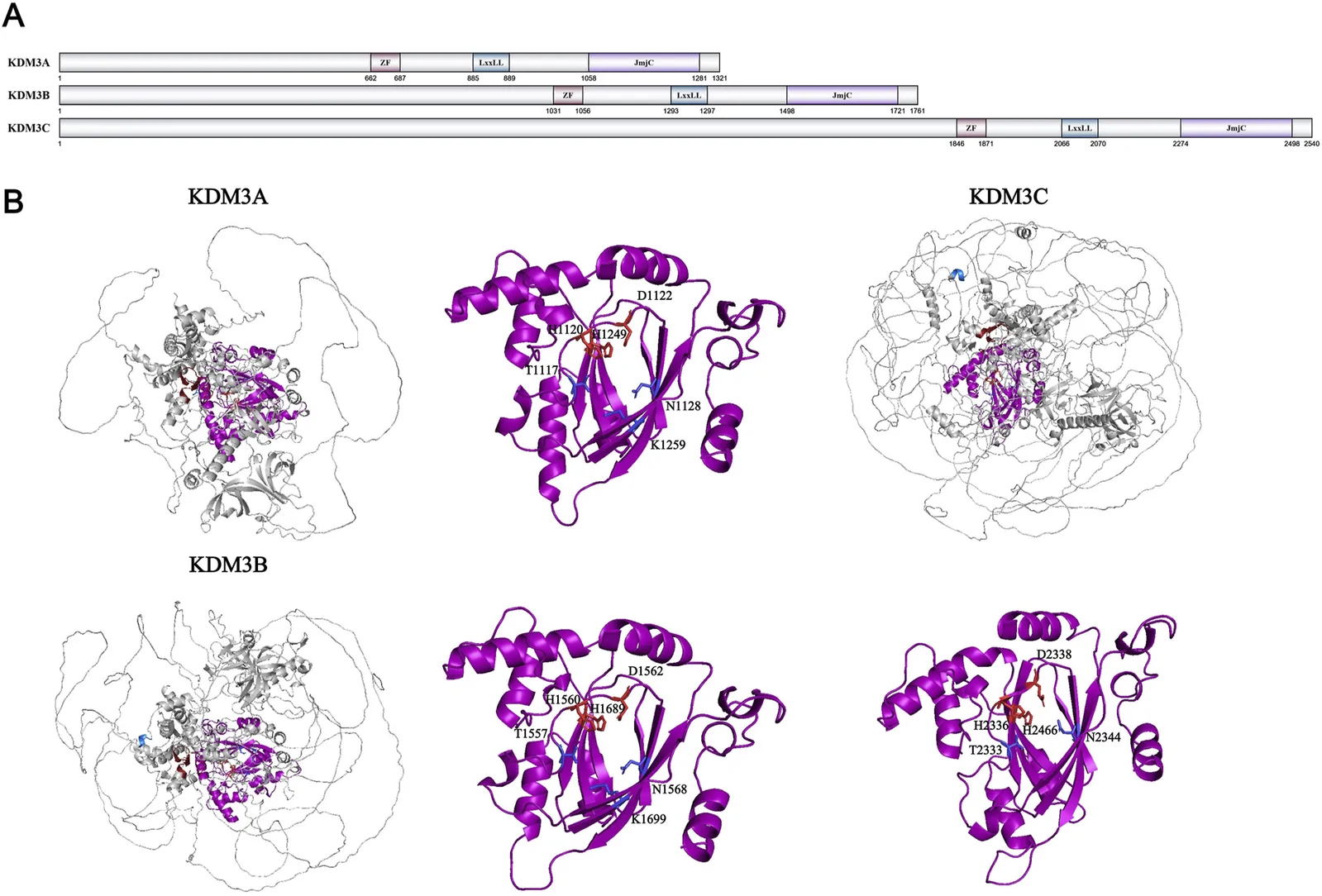
**(A)** Domains and sequences of KDM3 Family. The sequences of amino acids are annotated from UniProt database (KDM3A: Q9Y4C1; KDM3B: Q7LBC6; KDM3C: Q15652); **(B)** Structure of KDM3 family proteins from AlphaFold. The color of each structural domain corresponds to **(A)**. The amino acid required for Fe^2+^/α-KG binding sites are shown in red/blue respectively in stick form. The core pockets of KDM3B and KDM3C have been analyzed by X-ray (PDB ID: KDM3B-4C8D; KDM3C-2YPD). Only the core pocket of KDM3B is analyzed together with Fe^2+^ and α-KG. The core pocket structure analysis of KDM3A and KDM3C is based on AlphaFold simulation data and KMD3B data.

In addition to histones, the KDM3 family also exhibits demethylation activity towards some non-histone proteins. For example, KDM3A can inhibit the pro apoptotic function of p53 protein by removing the methyl modification on p53-K372 (Ramadoss et al., 2017). KDM3A can also remove methylation modifications on STAT3-K140 to regulate the JAK/STAT3 signaling pathway and inhibit the differentiation of immune B cells (Yin et al., 2022). KDM3C can regulate the methylation level of MDC1-K45 to regulate the DNA repair process. KDM3C can affect the methylation level of MDC1-K45 to regulate the DNA repair process mediated by RNF8 and BRAC1 (Watanabe et al., 2013). The function of KDM3A that has been extensively studied is that it can promote the expression of chromatin condensation related genes such as Tnp and Prm by removing H3K9 methylation on the promoters during sperm development (Okada et al., 2007; Liu Z. et al., 2010). Knockout of KDM3B/C in mice can also impair the development of male germ cells and lead to corresponding infertility in mice (Kuroki et al., 2013; Liu et al., 2015; Nakajima et al., 2016). Moreover, the expression of KDM3A upregulates the levels of multipotent transcription factors Oct4 and Sox2, affecting the pluripotency of embryonic stem cells (Ma et al., 2008; Zhu et al., 2021). Knockout of KDM3A/3B can lead to differentiation of embryonic stem cells and embryonic death (Kuroki et al., 2018). Finally, the KDM3 family also plays an important role in the occurrence of cancer. KDM3A activates transcription factors and upregulates their expression through its demethylase-dependent manner, thereby altering transcriptional levels of cancer-associated genes and promoting tumorigenesis in various cancers, including gastric and breast cancer (Ramadoss et al., 2017; Zheng J. et al., 2024; Fan et al., 2023). In contrast to the well-established oncogenic role of KDM3A, both KDM3B and KDM3C exhibit dual tumor-suppressive and tumor-promoting properties across different types of cancer. For example, KDM3B is highly expressed in prostate cancer and inhibits the progression of acute myeloid leukemia (AML) by regulating HOXA1 expression through affecting RARE transcription (Björkman et al., 2012; Xu et al., 2018). KDM3C can improve the sensitivity of breast cancer to ionizing radiation and PARP inhibitors, while promoting the development of AR-negative prostate cancer by inhibiting the TNF-α signaling pathway. The mechanism research of the KDM3 family in diseases is relatively complete, which can in turn promote the study of the basic functions and structures of the KDM3 family and provide assistance for the development of related targeted drugs.

### KDM4 family

In the same year as the KDM3 family discovery, Professor Yang Shi’s team identified the JMJD2/JHDM3/KDM4 family (with KDM4C also known as GASC1) as histone demethylases through systematic screening of known JmjC protein families. The KDM4 family comprises six members (KDM4A-F), among which KDM4E and KDM4F are potentially intronless pseudogenes, while KDM4A and KDM4C share higher sequence similarity compared to KDM4B and KDM4D (Watanabe et al., 2013; Katoh and Katoh, 2004; Yoshihama et al., 2021). Their activity assays on KDM4A-D confirmed this: all KDM4A-D members have K9me3 demethylation activity, but KDM4A and 4C also demethylate K36me3, and KDM4D demethylates K9me2 (Whetstine et al., 2006). In subsequent studies, it was also confirmed that the KDM4A-C family also demethylate H3K9me2 and H3K36me2 (Wissmann et al., 2007; Chen et al., 2006; Cloos et al., 2006; Hillringhaus et al., 2011). All KDM4 (specifically KDM4A-D) family proteins contain basic JmjC and JmjN domains. Except KDM4D, KDM4A-C also have two PHD zinc finger domains and two Tudor domains with nucleic acid recognition and binding functions (Whetstine et al., 2006) (Figure 7A). Therefore, KDM4D exhibits different structural and functional characteristics compared to the other three family members. KDM4A-C are widely expressed in normal human tissues, while KDM4D is only highly expressed in male testes (Labbé et al., 2013). Not only does KDM4D exhibit higher demethylation activity towards H3K9me2 compared to KDM4A-C, but it also demethylates H1.4K26me2/me3 (Trojer et al., 2009). Compared with the KDM3 family, the KDM4 family has been well characterized through crystallographic studies (Figure 7B). JmjC and JmjN domains together form a barrel-like catalytic core structure in three-dimensional space, which is necessary for KDM4 family‘s enzymatic function. Structural studies reveal that this barrel-like structure consists of two large β-sheets, each composed of four antiparallel β-strands (Chen et al., 2006; Ng et al., 2007). Taking KDM4A’s barrel structure as an example, Fe^2+^ ions will form coordination bonds with strictly conserved residues His188, Glu190, His276, and α-KG will form intermolecular forces such as hydrogen bonds with polar amino acids such as Tyr132, Asn198 and Lys206 to stabilize the catalytic structure (Chen et al., 2006; Ng et al., 2007). After that, the structure of KDM4A’s β-sheets will change and make the substrate enter the active pocket (Ng et al., 2007). This structural change is the fundamental basis for demethylase activity across all JmjC dioxygenase family members. For KDM4A, besides the amino acids in the core catalytic structure, amino acids such as Ile71 and Asn86 located in the adjacent domain could also affect its activity (Hillringhaus et al., 2011). Interestingly, Ser288 and Thr289 of KDM4A can affect the specificity of substrates. When they were mutated to the corresponding residues in KDM4D (S288A and T289I), KDM1A exhibits enhanced activity towards both H3K9me2 and H3K36me2 like KDM4D (Chen et al., 2006). For the substrate preference of KDM4A-C, the study found that the presence of two consecutive glycines at the ±1–2 site of methylated lysine can greatly improve the demethylation activity of enzymes (Chen et al., 2007). KDM4A-C also has two domains with recognition and binding functions, which is Tudor and PHD domains. Tudor domain can specifically bind histone modification sites such as H3K4me3, H3K23me3, H4K20me3 to promote the demethylation process on H3K9 or H3K36 (Huang et al., 2006; Horton et al., 2010; Su et al., 2016). It is only known that the PHD zinc finger domain on other proteins can recognize modifications such as methylation and acetylation of histones, as well as specific DNA sequences. However, the specific role of the PHD domain on KDM4A-C still needs to be studied. For KDM4D containing only the JmjC domain, research suggests that it mainly recognizes substrates through the internal structure of JmjC, rather than through the combined action of external and internal structures like KDM4A-C (Hillringhaus et al., 2011).

**FIGURE 7:**
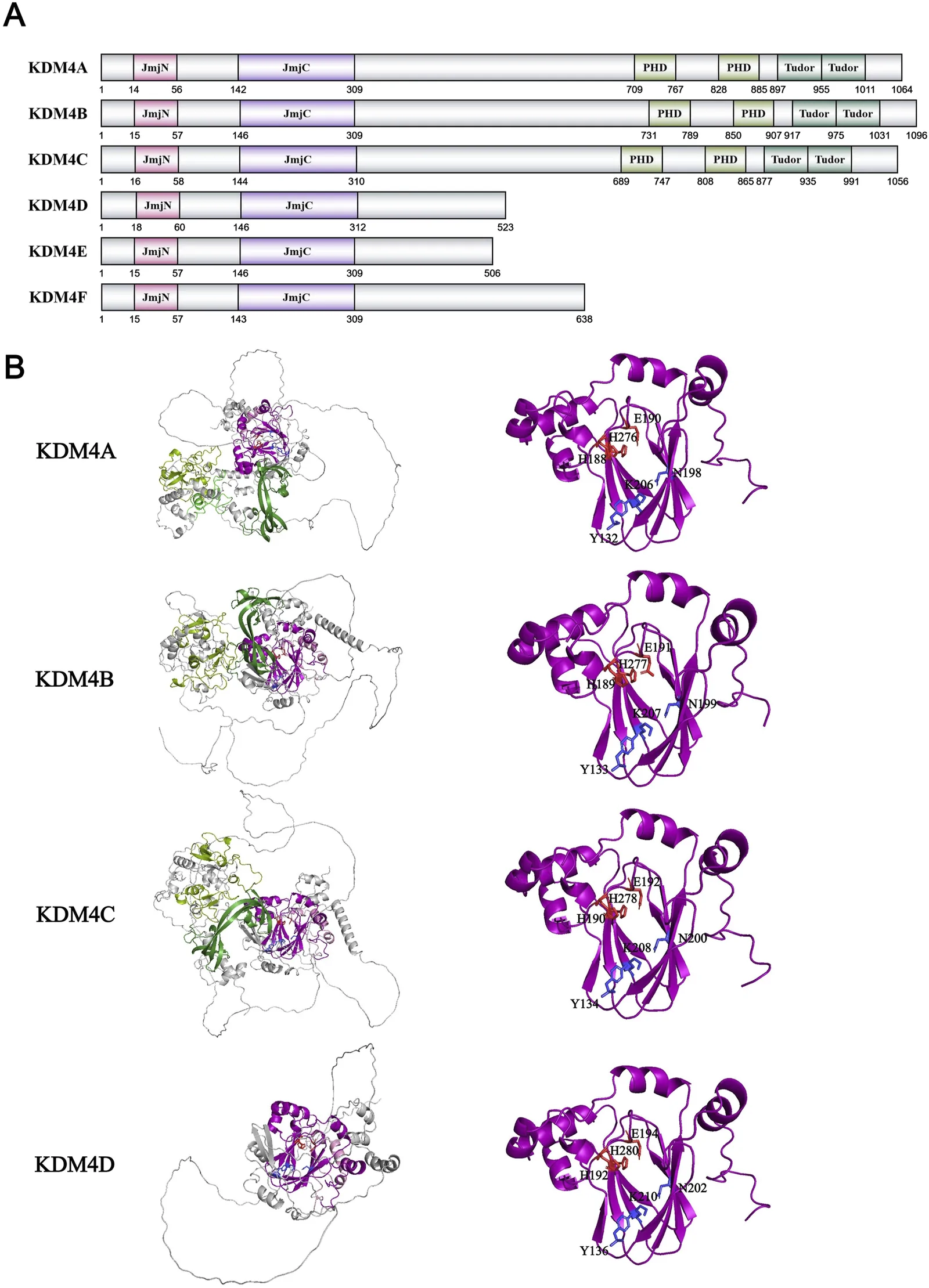
**(A)** Domains and sequences of KDM4 Family. The sequences of amino acids are annotated from UniProt database (KDM4A: O75164; KDM4B: O94953; KDM4C: Q9H3R0; KDM4D: Q6B0I6; KDM4E: B2RXH2; KDM4F: A0A1W2PPD8); **(B)** Structure of KDM4 family proteins from AlphaFold. The color of each structural domain corresponds to **(A)**. The amino acid required for Fe^2+^/α-KG binding sites are shown in red/blue respectively in stick form. Above proteins’ core pockets have been analyzed by X-ray (PDB ID: KDM4A-2GP5; KDM4B-4LXL; KDM4C-2XML; KDM4D-3DXU).

KDM4A-C can also demethylate non-histone substrates such as WIZ-K305me3, CDYL1-K135me3, G9a-K185me3, and multiple tri-methylation sites on CSB with varying efficiencies (Ponnaluri et al., 2009). These proteins are essential for H3K9 methylation to form inhibitory heterochromatin: WIZ stabilizes G9a/GLP heterodimers and activates H3K9 methyltransferase activity in G9a, while CDYL1 recognizes H3K9me3 to form local inhibitory chromatin structures (Shi et al., 2003; Ueda et al., 2006). This process suggests that the regulation of histone methylation is not a competitive modification of a single histone site, but a complex process that affects the upstream of the methylation modifying enzyme. KDM4C can also demethylate Pc2-K191me2 to activate the expression of E2F1, a downstream developmental gene (Yang et al., 2011). The KDM4 family plays an important role in many physiological functions. KDM4A-C can remove the inhibitory modification H3K9me3 to promote the expression of hematopoietic stem cell related genes such as Taf1b and Nom1 and maintain the stem and function of hematopoietic stem cells (Agger et al., 2019). KDM4A mediates M1 polarization of macrophages in multiple immune response mechanisms and regulates the expression of inflammatory cytokines in coordination with Wnt signaling pathway (Cai et al., 2023; Zheng L. et al., 2024; Zhang et al., 2024). In tumor cells, the inhibition of KDM4B will cause abnormal hypermethylation of the loci around the DNA break site, thus masking the local H3K9 trimethylation signal which is crucial to the DNA homology dependent repair pathway, and leading to the reduction of DNA repair factors (Sulkowski et al., 2020). The KDM4 family also exhibits functional heterogeneity in tumors. For example, in breast cancer, KDM4A activates Notch1-NICD signaling pathway in a demethylase-dependent manner to drive the development and metastasis of breast cancer, while KDM4B reduces the expression of PHGDH and inhibits the development and metastasis of breast in the same mechanism (Pei et al., 2023; Wang X-Y. et al., 2024). Compared to the KDM3 family, the research on the structure and function of the KDM4 family is more comprehensive, and inhibitory drugs and treatment plans targeting the KDM4 family have also made rapid progress and entered clinical trials.

### KDM5 family

The KDM5 family was discovered in 2007. Unlike the previous four KDM families discovered by a single team, the four members of the KDM5 family, KDM5A/JARID1A/RBP2, KDM5B/JARID1B/PLU-1, KDM5C/JARID1C/SMCX, and KDM5D/JARID1D/SMCY, were discovered and published by multiple research groups in just 1 month in March 2007. This also indicates that the research on histone demethylases has entered a period of rapid development. The team of Zhang Yi and William G. Kaelin Jr. initially discovered the high sequence similarity between the JmjC domains of KDM5 and KDM4 families and demonstrated the demethylation activity of KDM5A with H3K4me2/me3 *in vitro* and *in vivo* (Klose et al., 2007). Subsequently, the teams of Professor Shi Yang, Professor Zhang Yi, and Professor Kristian Helin independently demonstrated the activity of KDM5A-D in H3K4me2/me3 *in vitro* and *in vivo* (Yamane et al., 2007; Iwase et al., 2007; Christensen et al., 2007). KDM5 family members contain a set of JmjN-JmjC catalytic domains, an ARID domain, a C5HC2 Zn Finger domain, and multiple PHD zinc finger domains (KDM5A, B have three, KDM5C, D have two) (Figure 8A). With the help of X-ray crystallography, the core structure of KDM5A-C in the KDM5 family has been fully revealed. Firstly, like the KDM4 family, the JmjN and JmjC domains form a complete catalytic domain in the KDM5 family (Horton et al., 2016). The amino acids His, Glu/Asp bound to Fe^2+^ ions and Tyr, Ser, Asn, and Lys bound to α-KG acid in the catalytic domain are highly conserved, consistent with the entire JmjC family (Horton et al., 2016) (Figure 8B). The unique C5HC2 zinc finger domain of KDM5C is also indispensable. It forms a complete catalytic core together with the JmjN JmjC catalytic domain, but how the zinc finger domain specifically affects the catalytic process is still unknown (Yamane et al., 2007; Horton et al., 2016). The absence of ARID and PHD domains inserted between JmjN and JmjC has negligible effects on the *in vitro* enzymatic activity kinetics of KDM5A-C (Horton et al., 2016; Johansson et al., 2016). However, research has found that the PHD zinc finger domain in KDM5A/B has histone binding activity: the N-terminal PHD domain can recognize H3K4me0 (KDM5C/D can also recognize H3K4me0/me3, but with weaker binding ability), while the C-terminal PHD domain can recognize H3K4me0/me3(The PHD function in the middle is temporarily unknown) (Klein et al., 2014; Zhang et al., 2014). This recognition stabilizes the substrate protein binding conformation and improves catalytic efficiency when KDM5A/B performs demethylation function *in vivo* (Zhang et al., 2014; Longbotham et al., 2019). ARID is a type of structural domain that can recognize DNA sequences rich in AT bases. Research shows that the ARID domain on KDM5A/B can recognize CCGCCC and GCAC (A/C) sequences, but this binding is spatially mutually exclusive with the interaction between the N-terminal PHD domain and H3K4me0 (Horton et al., 2016; Scibetta et al., 2007; Tu et al., 2008). The existing structural and mechanical evidence of enzyme activity favor the combination of the latter, which is true. Therefore, the role of arid domain remains to be studied and discovered.

**FIGURE 8:**
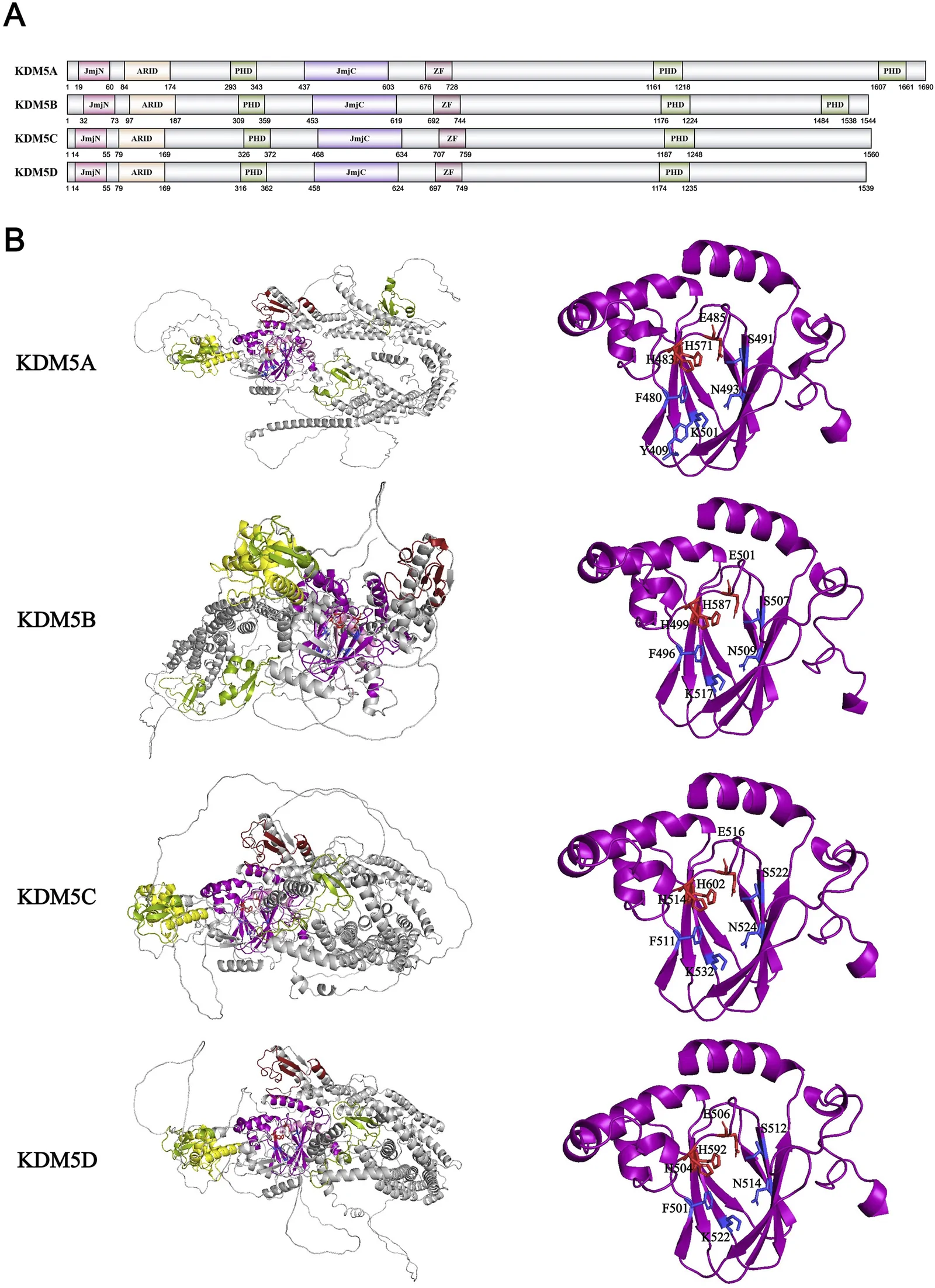
**(A)** Domains and sequences of KDM5 Family. The sequences of amino acids are annotated from UniProt database (KDM5A: P29375; KDM5B: Q9UGL1; KDM5C: P41229; KDM5D: Q9BY66); **(B)** Structure of KDM5 family proteins from AlphaFold. The color of each structural domain corresponds to **(A)**. The amino acid required for Fe^2+^/α-KG binding sites are shown in red/blue respectively in stick form. The core pockets of KDM5A, KDM5B and KDM5C have been analyzed by X-ray (PDB ID: KDM5A-5E6H; KDM5B-5A1F; KDM5C-5FWJ). KDM5D has no structural analysis on core pocket. The core pocket structure analysis of KDM5D is based on AlphaFold simulation data and KMD5A-C data.

Currently, only KDM5D has been found in the KDM5 family to remove the methylation of p38-K165 and inhibit its phosphorylation, thereby suppressing the MAPK signaling pathway (Chen et al., 2024). In order to find more non-histone substrates of the KDM5 family, researchers have developed a method based on systematic mutations of H3K4 adjacent amino acids to detect the relative activity of the KDM5 family and thus speculate on the non-histone substrates and sites of the KDM5 family (Hoekstra et al., 2022). By this way, the team detected several possible substrates of the KDM5 family. The KDM5 family can also interact with different proteins in a demethylase-independent mechanism, such as retinoblastoma protein (RB). RB is a gene that inhibits cancer development in most human cancers, and KDM5A/RBP2 was discovered and named after the screening of RB binding proteins (Defeo-Jones et al., 1991). KDM5A utilizes its LxCxE motif to interact with RB, while KDM5B, lacking this motif, uses its NTE1A region for RB binding (Benevolenskaya et al., 2005; Roesch et al., 2005). KDM5C/D, which differs significantly in structure from KDM5A/B, was not found to bind to RB. The binding of RB to KDM5A/B inhibits the Notch signaling pathway, maintains the expression of neuroendocrine transcription factor ASCL1, and promotes the development of small cell lung cancer tumors (Oser et al., 2019). RB can also localize KDM5A/B to RB target genes such as E2F and remove H3K4me3 modification to silence target genes, promoting the process of cell aging (Chicas et al., 2012). KDM5A/B are also components of histone deacetylase transcriptional repression complexes NuRD and SIN3B, while KDM5C prefers to bind to CoREST to affect protein expression and related signaling pathways (Pavlenko et al., 2022). Therefore, the KDM5 family plays an important role in various physiological functions such as cell aging, cell differentiation, and neural development. KDM5A is highly enriched in the promoter of preadipocytes and serves as a dual regulator of gene expression, which is necessary for promoting early differentiation and activation of cell cycle genes in proliferating cells (Brier et al., 2017). KDM5B suppress endogenous reverse transcription elements such as MMVL in a demethylase-independent manner by recruiting H3K9 methyltransferase SETDB1, thereby inhibiting cytoplasmic RNA and DNA sensing pathways and subsequent type I interferon response, allowing tumors to gain immune escape (Zhang et al., 2021). In autoimmune diseases, KDM5B can also inhibit the expression of NF-κB by removing the H3K4me3 modification on its promoter site, activating the NF-κB signaling pathway to promote the production of pro-inflammatory cytokines such as IL-1β, IL-6, and TNF-α macrophages (Zhang Y. et al., 2023). KDM5C affects neural development, anxiety, memory, cognition, and other behaviors by regulating the Wnt/β-catenin signaling pathway (Zhang Y. et al., 2023). The earliest disease discovered to be caused by abnormalities in the KDM5 family was X-linked intellectual disability caused by mutations in the KDM5C gene located on the X chromosome, which is speculated to be due to abnormalities in neuronal development and functional regulation caused by mutations in KDM5C (Jensen et al., 2005; Hatch and Secombe, 2022). In the study of prostate cancer cells resistant to docetaxel, KDM5D was found to interact with AR, inhibiting the activation of the AR pathway and the development of docetaxel resistance (Komura et al., 2016). KDM5A/B is considered as a kind of tumor promoting factor in the development of tumors. KDM5A activates the PTEN/Akt signaling pathway by upregulating ROCK1 in liver cancer, KDM5A activates the PTEN/Akt signaling pathway to make cancer achieve cisplatin tolerance by upregulating ROCK1 in liver cancer, while KDM5B can interact with SKP2 to activate the Akt signaling pathway, promoting the proliferation and migration of prostate cancer (Fang et al., 2023; Brown et al., 2024). KDM5C promotes the expression of SF3A3 and the development of bladder cancer by interacting with E2F6 to remove the H3K4me2/me3 modification at the SF3A3 promoter (Liu et al., 2022). In contrast, KDM5D can inhibit the MAPK signaling pathway and cancer development by suppressing the methylation and phosphorylation of p38 (Chen et al., 2024). In summary, there are numerous and relatively complete studies on the basic functions and applications of the KDM5 family in diseases.

### KDM6 family

Similar to the KDM5 family, various members of the KDM6 family were also identified by multiple research groups in the second half of 2007. Professor Kristian Helin’s team was the first to identify that the KDM6 family has H3K27me3 demethylation activity. Previous studies have shown that the methyltransferase complex PRC2 in embryonic stem cells can increase the level of H3K27me3, thereby inhibiting cell development genes and maintaining cell stemness (Boyer et al., 2006; Bracken et al., 2006; Lee et al., 2006; Surani et al., 2007). Therefore, in order to explain the rapid changes in H3K27me3 levels during subsequent embryonic development and stem cell differentiation, they searched for highly expressed protein families containing the JmjC domain in embryonic stem cells. Finally, they discovered and confirmed that two members of the KDM6 family, KDM6A/UTX and KDM6B/JMJD3, can remove the methylation modification of H3K27me2/me3 (Agger et al., 2007). Shortly thereafter, multiple teams also confirmed this discovery (De Santa et al., 2007; Xiang et al., 2007; Hong et al., 2007). Although another member of the KDM6 family, KDM6C/UTY, exhibits high homology with KDM6A/UTX, it was found in subsequent experiments that UTY has almost no demethylation ability *in vitro* (Lan et al., 2007; Shpargel et al., 2012). The KDM6 family collectively contains a JmjC domain with similar sequences and a zinc finger domain. KDM6A and KDM6C have a higher degree of homology, and they also possess a set of tetrapeptide repeat domains TPR (Figure 9A). The TPR domain has a conserved and unique spatial structure, which can mediate protein interactions and assembly of protein complexes. In addition, other structures such as a helical domain and a novel zinc finger domain were identified in the structural analysis of KDM6A, which can specifically recognize the spatial structure of substrates and bind to them (Sengoku and Yokoyama, 2011). Similar to the known JmjC family, the JmjC domain of KDM6A is also a barrel-like structure composed of 8 β-layers that are antiparallel (Figure 9B). The amino and carbonyl groups on the H3K27 main chain will form hydrogen bonds with Leu1127 and Gln1133 on the outer side of the KDM6A barrel structure to stabilize the protein structure (Sengoku and Yokoyama, 2011). Inside the catalytic center, the hydrogen bond structure pattern formed by KDM6A and the three methyl groups of K27me3 is similar to that of SET-domain methyltransferase and KDM4A: the hydroxyl group of Tyr1135, the carbonyl group of Gly1128, and the carboxyl group of Glu1148 (corresponding to Tyr177, Gly170, and Glu190 of KDM4A) respectively form characteristic hydrogen bonds with the methyl group of K27me3 to adjust the position of the methyl group and promote the reaction (Sengoku and Yokoyama, 2011). In addition, although H3K27 and H3K9 have the same A-R-Kme-S amino acid sequence, KDM6A can recognize the H3K27 site by specifically binding to different H3 fragments (such as H3Leu20-Thr32) through the outer layer structure of JmjC and new zinc finger structure (Sengoku and Yokoyama, 2011). The crystal structure analysis of KDM6B confirms that its substrate recognition mechanism is the same as that of KDM6A (Jones et al., 2018). Similarly, structural studies on KDM6C have confirmed that the KDM6 family is highly conserved in recognizing α-KG and Fe^2+^, but the Pro1214 mutation in the core domain of KDM6C (corresponding to Ile of KDM6A/B) significantly reduces its demethylation activity (Walport et al., 2014). However, when KDM6A is inactivated during embryonic development, KDM6C, as a homologous protein of KDM6A on the Y chromosome, has a compensatory effect on KDM6A (Shpargel et al., 2012). Combined with KDM6A being one of the few proteins on the X chromosome that escape X inactivation, it is evident that KDM6A/C plays an important role in embryonic development beyond histone demethylation (Shpargel et al., 2012; Greenfield et al., 1998).

**FIGURE 9:**
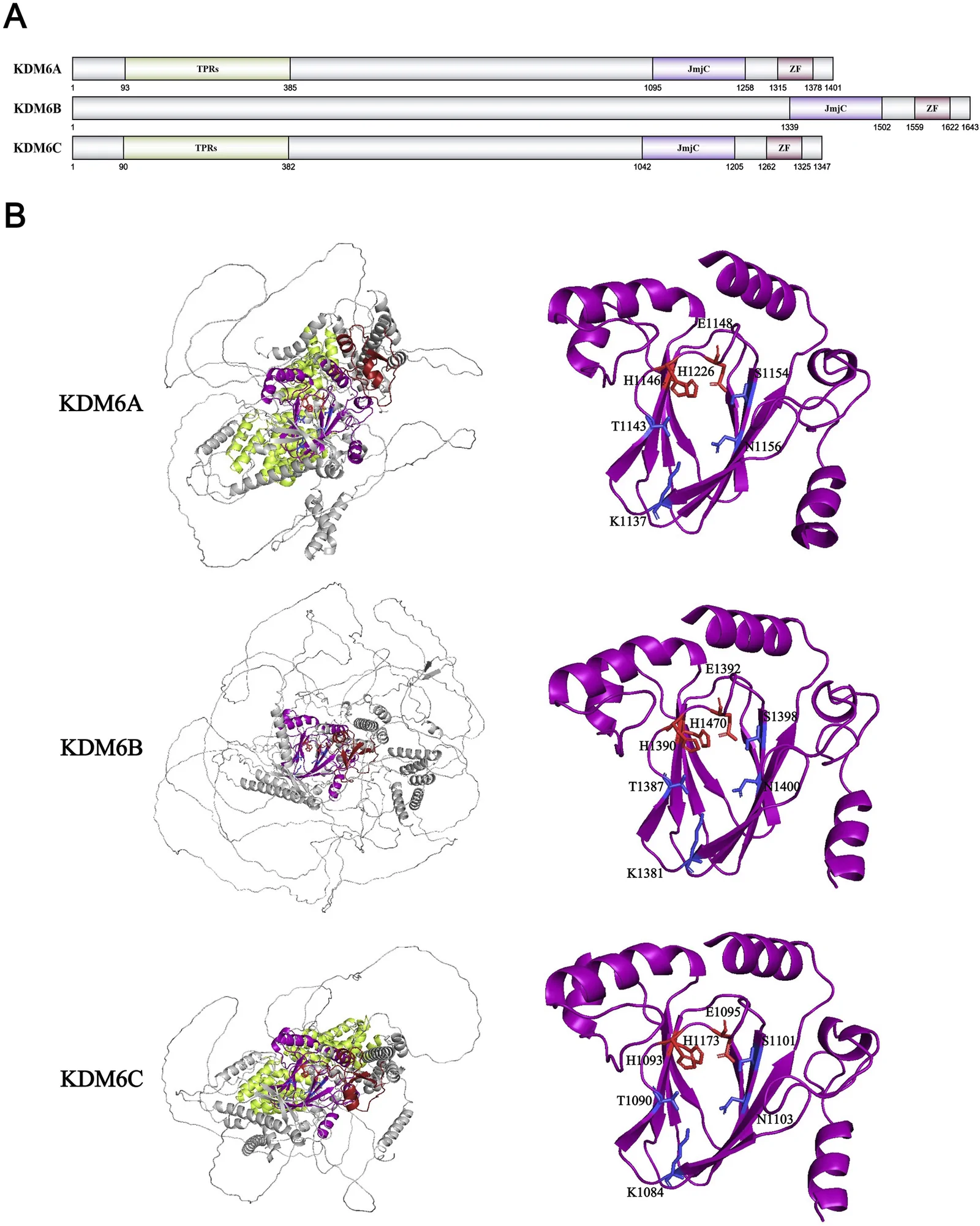
**(A)** Domains and sequences of KDM6 Family. The sequences of amino acids are annotated from UniProt database (KDM6A: O15550; KDM6B: O15054; KDM6C: O14607); **(B)** Structure of KDM6 family proteins from AlphaFold. The color of each structural domain corresponds to **(A)**. The amino acid required for Fe^2+^/α-KG binding sites are shown in red/blue respectively in stick form. Above proteins’ core pockets have been analyzed by X-ray (PDB ID: KDM6A-3AVR; KDM6B-2XUE; KDM6C-3ZLI).

In the study of non-histone substrates, the KDM6 family, like the KDM5 family, has received little research. Until now, only KDM6B has been found to demethylate RB-K810me, thereby reducing the phosphorylation level of RB and promoting the aging of WI38 cells and the formation of age-related heterochromatin lesions (Zhao et al., 2015). In addition to RB, the KDM6 family can also interact with different proteins. For example, KDM6B can be recruited by p53 through interactions and jointly act on the promoters and distal enhancers of downstream genes of p53 after DNA damage (Williams et al., 2014). KDM6A can also recruit different epigenetic modifying enzymes such as KMT2D and P300 to interact with each other to form a complex and add the active modification (H3K4me1 and H3K27Ac) to the enhancer, thus inhibiting the development of cancer and promoting the differentiation of embryonic stem cells (Wang et al., 2017; Shi et al., 2021). KDM6A can also be used as a component of COMPASS complex to regulate aging genes in mice (Sera et al., 2021). In addition, KDM6 family is highly expressed in many stem cells (hematopoietic stem cells, embryonic stem cells, etc.) and regulates their differentiation and development (Cabrera Zapata et al., 2021; Kobori et al., 2023; Wang J. et al., 2024; Zhang et al., 2025). For example, in muscle stem cells, KDM6B can activate the synthesis of hyaluronic acid and promote the regeneration of muscle cells by removing the H3K27me3 marker on Has2 gene (Nakka et al., 2022). Loss of KDM6A in neural stem cells results in impaired brain and neuronal development, resulting in infant mortality (Koizumi et al., 2022; Chen et al., 2025). It is interesting that in early pregnancy of mice, KDM6B in their uterine fibroblasts can affect gene expression in late pregnancy by regulating the level of H3K27me3, thereby affecting delivery time (McIntyre et al., 2025). KDM6C, as a downstream effector protein of the NKX3.1-G9a pathway, is an important marker of NKX3.1 promoting male prostate differentiation (Dutta et al., 2016). In multiple myeloma, KDM6A can induce immune escape and drug tolerance in cancer cells by regulating immune related proteins such as CD38/48 (Dupéré-Richer et al., 2024; Liu et al., 2024). In cancer research, KDM6A/B is typically heterogeneous. For example, in lung cancer, KDM6A inhibits tumorigenesis by suppressing the level of H3K27me3 methyltransferase EZH2, but KDM6A also collaborates with KMT2B to abnormally activate the Wnt pathway and promote cancer development (Wu et al., 2018; Leng et al., 2020). KDM6B in acute myeloid leukemia also experiences the same situation (Yu et al., 2018; Li et al., 2018; Mallaney et al., 2019). KDM6C has been less studied in cancer research independently, typically being investigated alongside its homolog KDM6A (Andricovich et al., 2018). Not only in cancer research, but KDM6C has also received less attention in broader scientific research. In summary, within the KDM6 family, KDM6A/B have been extensively characterized in terms of their functions and disease-related mechanisms, while KDM6C’s biological roles and molecular mechanisms remain to be elucidated.

### KDM7 family

Compared with the previous KDM family, the KDM7 family was discovered later. In 2010, the first member of the KDM7 family, KDM7B/PHF8, was identified by Professor Christopher J. Schofield’s team as possessing demethylase activity towards three histone methylation substrates: H3K9me1/me2 (Loenarz et al., 2010). Subsequently, KDM7A/KIAA1718 was demonstrated to possess H3K9me1/me2 and H3K27me1/me2 demethylase activity, while KDM7C/PHF2 was shown to possess H3K9me1 demethylase activity (Huang et al., 2010; Tsukada et al., 2010; Wen et al., 2010). Meanwhile, KDM7B/PHF8 has also been demonstrated to possess demethylation activity of H4K20me1 (Qi et al., 2010; Liu W. et al., 2010). The KDM7 family contains a JmjC domain and a PHD zinc finger domain (Figure 10A). The PHD domains of KDM7B and KDM7C can stabilize binding conformations through hydrophobic interactions and CH-π bonds formed between residues such as Tyr and Met with H3K4me2/me3 methyl groups, thereby enhancing catalytic efficiency towards H3K9me1/me2 (Horton et al., 2010; Wen et al., 2010; Feng et al., 2010) (Figure 10B). In contrast, the greater spatial separation between the PHD and JmjC domains in KDM7A (attributed to a longer and more rigid linker) competes with histone binding due to limited histone tail length, resulting in reduced catalytic efficiency towards H3K9me2 (Horton et al., 2010). Like previously characterized KDM families, the JmjC domains of the KDM7 family maintain highly conserved residues for coordinating Fe^2+^ and α-KG cofactors (Yu et al., 2010; Horton et al., 2011). The di-methyl substrate specificity of KDM7A/B primarily results from their compact catalytic centers (KDM7B: 169.10 Å vs. KDM4A: 722.36 Å), with peripheral aromatic residues such as Phe250 and Tyr234 further restricting tri-methyl group access (Yu et al., 2010; Horton et al., 2011). Additionally, KDM7C’s His335 (corresponding to Asn333 in KDM7B) possesses a larger, more rigid imidazole group that likely restricts di-methyl group access and binding, limiting its substrate specificity to K9me1 (Horton et al., 2011). Interestingly, while KDM7C alone lacks K9me2 activity, phosphorylation by protein kinase A enables its interaction with DNA-binding protein ARID5B, facilitating ARID5B-K336me2 demethylation and revealing latent H3K9me2 demethylase activity (Baba et al., 2011). Furthermore, some studies have reported KDM7C’s ability to demethylate H4K20me3 *in vitro*, though this finding lacks supporting evidence from peptide, histone, and *in vivo* studies, and contradicts most previous research (Stender et al., 2012).

**FIGURE 10:**
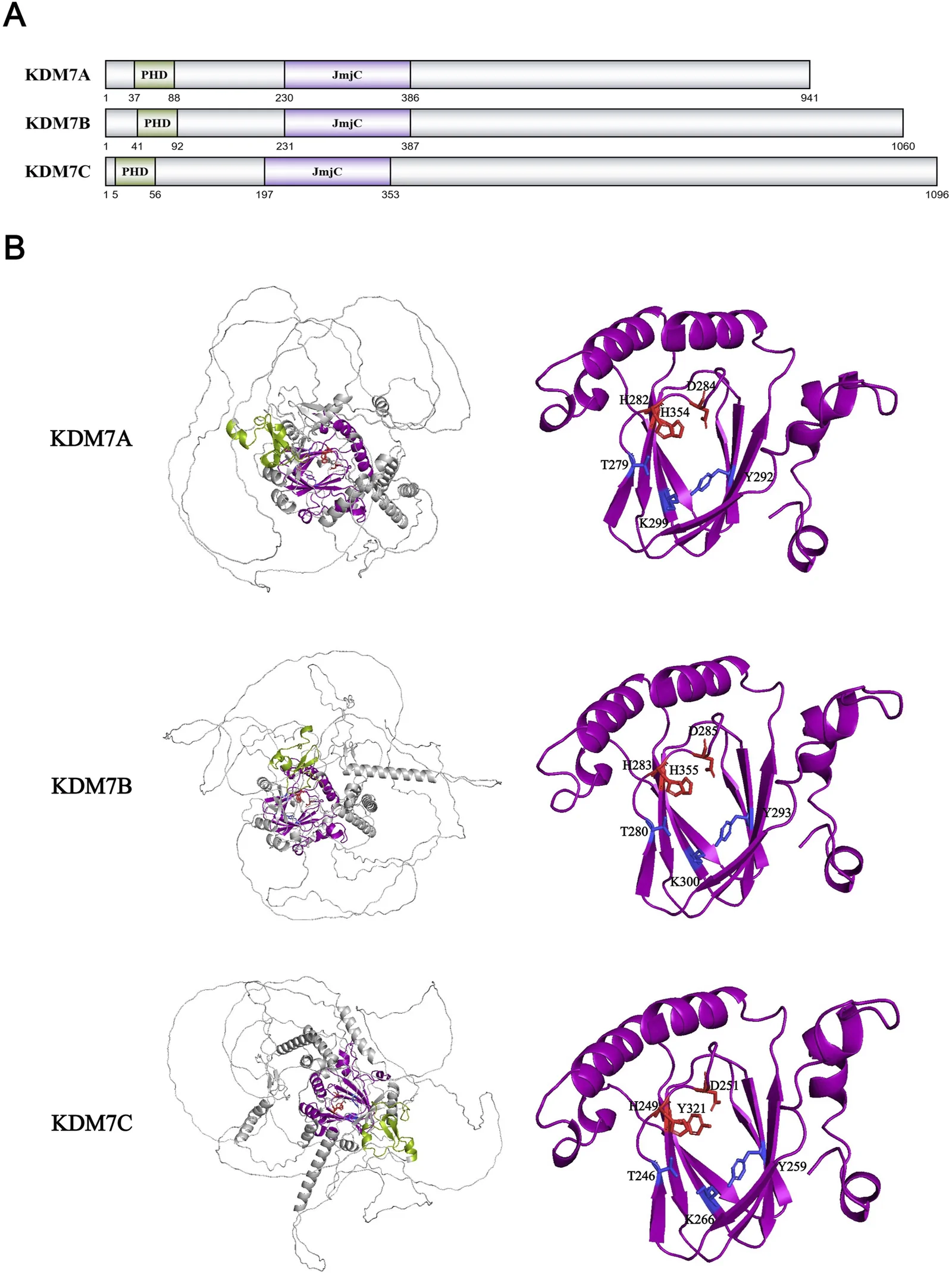
**(A)** Domains and sequences of KDM7 Family. The sequences of amino acids are annotated from UniProt database (KDM7A: Q6ZMT4; KDM7B: Q9UPP1; KDM7C: O75151); **(B)** Structure of KDM7 family proteins from AlphaFold. The color of each structural domain corresponds to **(A)**. The amino acid required for Fe^2+^/α-KG binding sites are shown in red/blue respectively in stick form. Above proteins’ core pockets have been analyzed by X-ray (PDB ID: KDM7A-3KV5; KDM7B-3K3O; KDM7C-3PU3).

Regarding non-histone substrates, KDM7A interacts with JAK2 and STAT1 to remove their Kme1/me2 modifications (specific sites unknown), thereby remodeling the immune microenvironment through the IFN-γ/JAK2/STAT1 signaling pathway (Yang et al., 2023). KDM7B interacts with TOPBP1 and demethylate TOPBP1-K118me1 to activate ATR1 and regulate DNA damage repair processes (Ma et al., 2021). KDM7B also interacts with YY1 to enhance transcription of electron transport chain genes, generating mitochondrial reactive oxygen species that drive cancer progression (Wu et al., 2024). KDM7C interacts with cohesin subunit RAD21, localizing the cohesin complex to topologically associated domains and chromatin loops in a demethylase-independent manner to maintain DNA replication in mouse neural stem cells (Feng et al., 2024). Moreover, the KDM7 family plays indispensable roles in various physiological processes, including neural system development and memory formation (Qi et al., 2010; Chen et al., 2018; Pappa et al., 2019; Wang et al., 2023), skeletal development and osteogenic differentiation (Kim et al., 2014; Pan et al., 2022; Shan et al., 2024). Surprisingly, while KDM5B promotes rDNA transcription, KDM6C competes for binding sites through its PHD domain, recruits H3K9me2/me3 methyltransferase SUV39H1, and suppresses rDNA transcription along with downstream genes OCT4 and KLF4 (Shi et al., 2014). This not only suggests demethylase-independent functions within the KDM7 family but also demonstrates potential functional antagonism among family members, necessitating case-specific analysis. Located on the X chromosome alongside KDM5C and KDM6A, KDM7B was associated with X-linked intellectual disability and cleft lip/palate through mutation studies prior to its demethylase activity identification (Laumonnier et al., 2005; Abidi et al., 2007). KDM7 family members exhibit diverse characteristics across different cancer types. KDM7A expression is reduced in liver cancer but elevated in pancreatic and breast cancers. KDM7A expression is reduced in liver cancer but promotes breast cancer progression by upregulating stemness-related factors KLF4 and c-MYC, along with anti-apoptotic factor Bcl-2, to inhibit apoptosis (Meng et al., 2020; Qu et al., 2022). KDM7B interacts with c-Jun to directly regulate cell migration-related genes including PRKCA (encoding PKCα) and downregulates the PTEN pathway, promoting proliferation and migration in HER2-negative gastric cancer (Tseng et al., 2020). In contrast to KDM7B, KDM7C functions as a p53-associated tumor suppressor (Lee et al., 2015). In liver cancer, palmitoylation enhances KDM7C’s E3 ubiquitin ligase activity towards SREBP1c, destabilizing SREBP1c and reducing SREBP1c-dependent lipogenesis, thereby inhibiting tumor growth (Jeong et al., 2023). Finally, despite being discovered relatively recently and having fewer overall studies, research on the KDM7 family has achieved relatively comprehensive understanding of its fundamental structure, functions, and roles in disease pathogenesis.

### Protein families containing the JmjC domain and other associated groups

Beyond KDM2-7, the JmjC domain-containing protein classification includes a unique family of small proteins exclusively featuring the JmjC domain, encompassing JMJD4-8, RIOX1/2, HIF1AN, HSPBAP1, and others. Most of them are under 100 kDa (with HR being an exception at 130 kDa). The majority lack identified histone demethylase activity, and even for those with reported activity like JMJD5, ongoing debates persist regarding the authenticity of their enzymatic function. JMJD5, identified in 2010 as having H3K36me2 demethylase activity and designated KDM8 (Hsia et al., 2010), failed to replicate this activity in subsequent *in vitro* and *in vivo* experiments (Del Rizzo et al., 2012; Youn et al., 2012; Wang et al., 2013; Wilkins et al., 2018). Structural analysis comparing JMJD5 with KDM7A/B revealed that side chains of Gln275, Trp310, and Trp414 obstruct H3K36me access to the catalytic center (Del Rizzo et al., 2012; Wang et al., 2013) (Figure 11A). Furthermore, JMJD5 has been proposed as an arginine C-3 hydroxylase, though this claim is currently supported only by structural analysis and *in vitro* peptide reactions, lacking histone/nucleosome and *in vivo* experimental evidence (Wang et al., 2013; Wilkins et al., 2018). Interestingly, JMJD5 and JMJD7 specifically recognize and bind histone tails containing methylated arginine residues such as H3R2 and H4R3, subsequently removing methylated arginine through cleavage to regulate transcription (Shen et al., 2017; Liu et al., 2017; Liu et al., 2018). This suggests alternative pathways for histone demethylation beyond enzymatic demethylation at specific sites. RIOX1/NO66 and RIOX2/MINA53 are the same as JMJD5. RIOX1 is demonstrated to possess demethylase activity towards H3K4me1/me2/me3 and H3K36me2/me3, while RIOX2 possesses H3K9me3 demethylase activity (Lu et al., 2009; Sinha et al., 2010). Correspondingly, subsequent *in vitro* and *in vivo* experiments have failed to replicate the demethylase activity of RIOX1/2 (Ge et al., 2012). Crystallographic analysis of RIOX1/2 also contradicts their function as demethylases, as histidine residues block histone lysine side chain access to the active site, and their JmjC domains lack two flexible loops conserved in KDM families that interact with histone methylation side chains (Chowdhury et al., 2014) (Figure 11B). These factors cast significant doubt on the existence of RIOX1/2 demethylase activity, highlighting the need for further experimentation and research to verify whether these small protein families genuinely possess histone demethylase activity.

**FIGURE 11:**
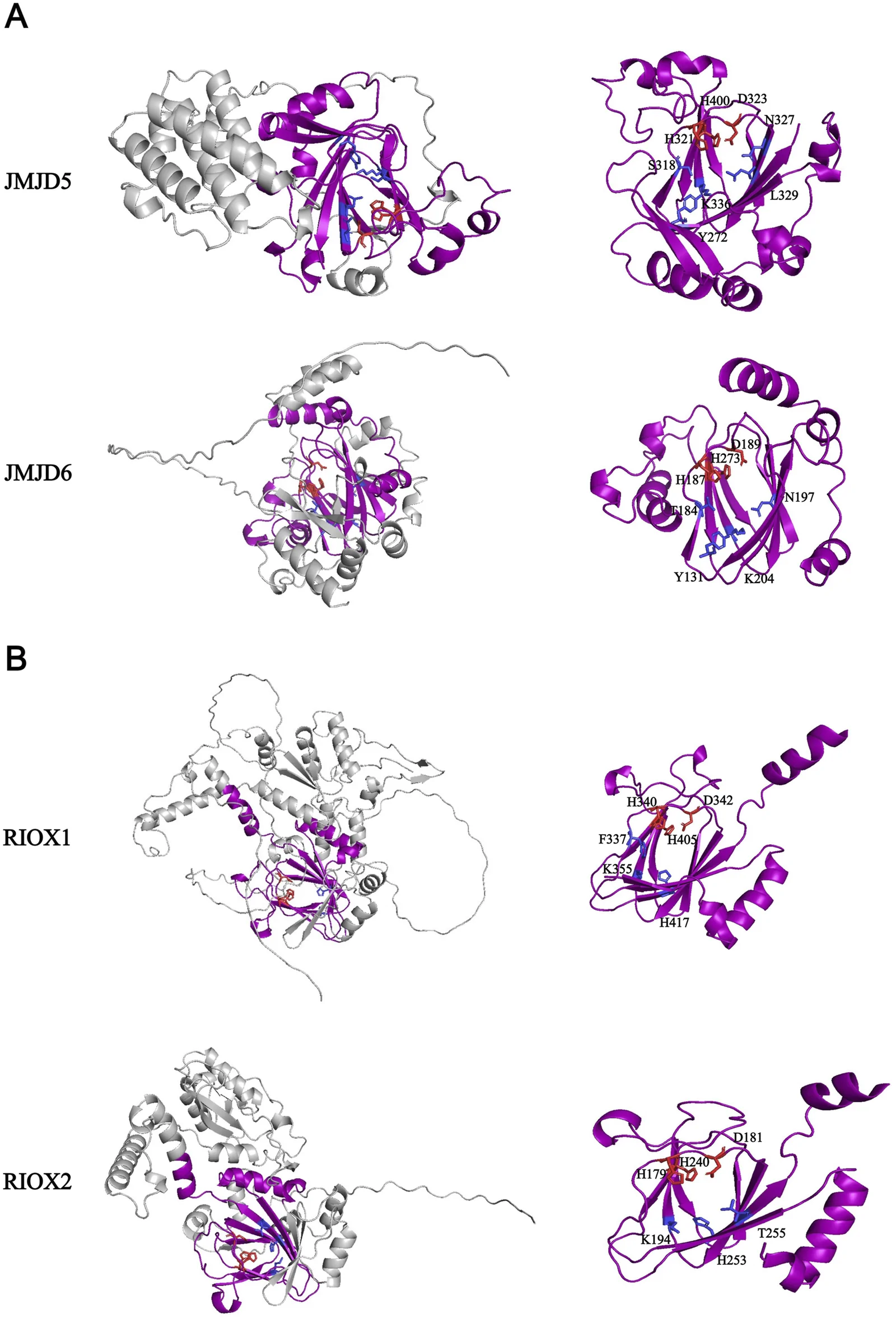
**(A)** Structure of JMJD5 and JMJD6. The sequences of amino acids are annotated from UniProt database (JMJD5: Q8N371; JMJD6: Q6NYC1). Above proteins’ core pockets have been analyzed by X-ray (PDB ID: JMJD5-4AAP; JMJD6-6GDY); **(B)** Structure of RIOX1 and RIOX2. The sequences of amino acids are annotated from UniProt database (RIOX1-Q9H6W3; RIOX2-Q8IUF8). Above proteins’ core pockets have been analyzed by X-ray (PDB ID: RIOX1-4CCM; RIOX2-4BU2). All structural data are from AlphaFold. The amino acid required for Fe^2+^/α-KG binding sites are shown in red/blue respectively in stick form. The purple domain in **(A)** and **(B)** is JmjC domain.

Except identified JmjC domain-containing proteins, recent studies have revealed that RAD23A/B (also known as HR23A/B), featuring a JmjC-like domain, can remove methylation modifications from H4K20me1/me2/me3 (Cao et al., 2020). This protein family, involved in DNA excision repair, consists of a ubiquitin-like (UBQ) domain, a ubiquitin-associated (UBA1/2) domain, and an XPC domain. Through construction of individual domain-deletion truncation mutants, researchers found that two UBA domains containing Fe^2+^-binding motifs within the JmjC-like domain are crucial for recognizing H4K20 and facilitating demethylation (Cao et al., 2020). Interestingly, while RAD23A/B exhibits activity towards all three H4K20 methylation states *in vitro*, ChIP-seq analysis reveals *in vivo* activity only at H4K20me1/3 sites (Cao et al., 2020).

### Progress in identifying H3K79 demethylases and histone arginine demethylases

Following the identification of demethylases for most histone methylation sites, the demethylase(s) for H3K79me1/me2/me3 remain(s) to be conclusively identified. Unlike other histone methylation sites, H3K79 is not located on the histone H3 tail but rather on an α-helix of H3 that interacts with H4, forming hydrogen bonds with the carbonyl group of H4R78’s backbone (White et al., 2001). Consequently, DOT1L, the only H3K79 methyltransferase identified to date, exhibits unique characteristics compared to other methyltransferases. First, DOT1L does not rely on the SET domain (used by most methyltransferases like EZH2 and SUV39H1 for catalysis) but instead utilizes a unique DOT1 domain for methyltransferase activity (Feng et al., 2002). Second, DOT1L cannot methylate free H3K79 and requires recognition of intact nucleosome structures to catalyze methylation (van Leeuwen et al., 2002; Valencia-Sánchez et al., 2019; Anderson et al., 2019; Worden et al., 2020). The methylation process of H3K79 suggests that its demethylation would significantly differ from that of methylated lysine sites located on histone tails, potentially requiring nucleosome structure recognition to facilitate the demethylation reaction. Finally, although some research teams have identified KDM2B as a potential H3K79me1/me2/me3 demethylase, this finding has not been replicated by other experimental groups (Kang et al., 2018).

In contrast to lysine, research on arginine demethylases has faced significant challenges. Similar to lysine methyltransferases, the arginine methyltransferase PRMT family was discovered as early as the last century (Lin et al., 1996; Tang et al., 1998). Due to arginine’s capacity for both symmetric and asymmetric dimethylation, the PRMT family is divided into three classes: Type I PRMTs (e.g., PRMT1/2/3) catalyzing asymmetric dimethylation (Rme2a), Type II PRMTs (e.g., PRMT5/9) catalyzing symmetric dimethylation (Rme2s), and PRMT7, which only catalyzes monomethylation (Rme1) (Blanc and Richard, 2017). Similar to the H3K79 site, arginine still lacks a universally recognized demethylase. *In vitro* peptide-level experiments suggest that arginine demethylation may also depend on the JmjC domain, and it is possible that KDM family members *in vivo* could perform arginine demethylation under the influence of different cofactors (Walport et al., 2016; Bonnici et al., 2023). Although the small JmjC domain-containing protein JMJD6 was identified in 2007 as having demethylase activity towards H3R2me1/me2 and H4R3me1/me2 (without specifying symmetric or asymmetric dimethylation) (Chang et al., 2007), most subsequent replication attempts have failed (Hong et al., 2010; Hahn et al., 2010; Han et al., 2012; Liu et al., 2013). Recent studies have revealed that another small JmjC domain-containing protein, RIOX2/MINA53, exhibits demethylase activity towards H4R3me2a and p53-R337me2a (Zhou et al., 2024; Zhou et al., 2025). Whether this finding can be replicated by other research teams remains to be seen. Except, conventional demethylation, there is also a protein peptidyl arginine deiminase 4 (PADI4) that can oxidize methylated arginine to methylated citrulline and then remove it (Wang et al., 2004; Cuthbert et al., 2004). Since this method has changed substrate and consumes a lot of energy, it is generally not included in the scope of arginine demethylation.

In conclusion, it is difficult to find the demethylation of H3K79 and histone arginine for different reasons. H3K79 has a special positional structure, and the corresponding demethylated protein may need a special structure to recognize or open nucleosomes to demethylate H3K79me. Arginine and lysine may share the same reaction system for demethylation. This makes it possible that arginine and lysine compete with each other for demethylation active sites, leading to weak or even no reaction of arginine demethylation. Nevertheless, the discovery of H3K79 and histone arginine demethylation still achieved significant results.

## The clinical significance and prospects of the demethylases

Since the discovery of the first demethylase, research on applying demethylases to clinical practice has commenced and achieved significant progress. Among them, the KDM1 and KDM4 families play significant roles in tumor progression and signal pathway regulation. Here, we will briefly introduce the targeted drug development status and application prospects of KDM1A and the KDM4 family.

KDM1A was the first discovered histone demethylase, hence the development of its related targeted drugs began relatively early. The inhibitor TCP (also known as Parnate), which targets the KDM1A homolog monoamine oxidase (AOL), was among the earliest inhibitors developed to suppress KDM1A. But obviously, TCP has a higher affinity with AOL. Consequently, subsequent research teams have used TCP as a core scaffold to further develop drugs with greater affinity for KDM1A. With ongoing research, more diverse types of KDM1A inhibitors have been identified. To date, KDM1A inhibitors that have entered clinical trials include Iadademstat (ORY-2001), CC-90011, IMG-7289, among others, with the highest progress currently reaching Phase II clinical trials. Additionally, KDM1A inhibitors have been explored in combination therapies with other drugs for cancer treatment. In a 2024 study evaluating Iadademstat combined with azacitidine for newly diagnosed AML patients, the combination therapy demonstrated promising efficacy against AML (Salamero et al., 2024).

The KDM4 family, as one of the earliest JmjC family proteins to have its structure elucidated, also had early development of related targeted drugs. Most small molecule inhibitors are α-KG analogs and their derivatives designed for the active pocket of KDM4, which inhibit KDM4 activity by chelating Fe^2+^ in the catalytic binding pocket. But most of these inhibitors cannot correctly recognize KDM4 family proteins and cannot penetrate the cell membrane. Therefore, although many alternative drugs have been developed, currently only TACH101 has entered clinical trials. TACH101 is an α-KG competitive KDM4 inhibitor that has entered phase I clinical trials (Chandhasin et al., 2023).

In addition to the aforementioned drugs, other families of drugs such as the KDM5 family inhibitor CPI-455 and the KDM6 family inhibitor GSK-J4 are in the pre-clinical research stage. Overall, the development of KDM family related inhibitors is still in the early stages and has broad prospects for related applications.

## Conclusion

Since the discovery of the first histone demethylase in 2004, significant progress has been made in the research of demethylase. So far, eight protein family members have been proved to have histone demethylation activity in human body. These proteins are distributed not only on the autosomal but also on the sex chromosome. In addition to their demethylation active sites, different subtypes and modifications of these proteins, such as variable shear, phosphorylation modification, and the formation of complexes with different proteins can change their active sites (Wissmann et al., 2007; Wang et al., 2015; Baba et al., 2011; Metzger et al., 2005). This indicates that the recognition sites of histone demethylase are affected by many factors, that is, the modification or mutation of important sites and the change of physiological environment will lead to the change of the spatial structure and active sites of histone demethylase. Epigenetics, represented by methylation, can rapidly affect gene transcription, protein expression and signal transduction of signaling pathways, which makes demethylases can regulate embryonic development, cell proliferation, neural differentiation and memory formation and other important physiological processes by affecting the methylation of histone and non-histone proteins (Figure 12) (Kouzarides, 2007). Consequently, notwithstanding the inherent complexities and methodological challenges associated with such investigations, the elucidation of demethylase discovery, structural characterization, functional analysis, and mechanistic studies will progressively emerge as a paramount research focus.

**FIGURE 12:**
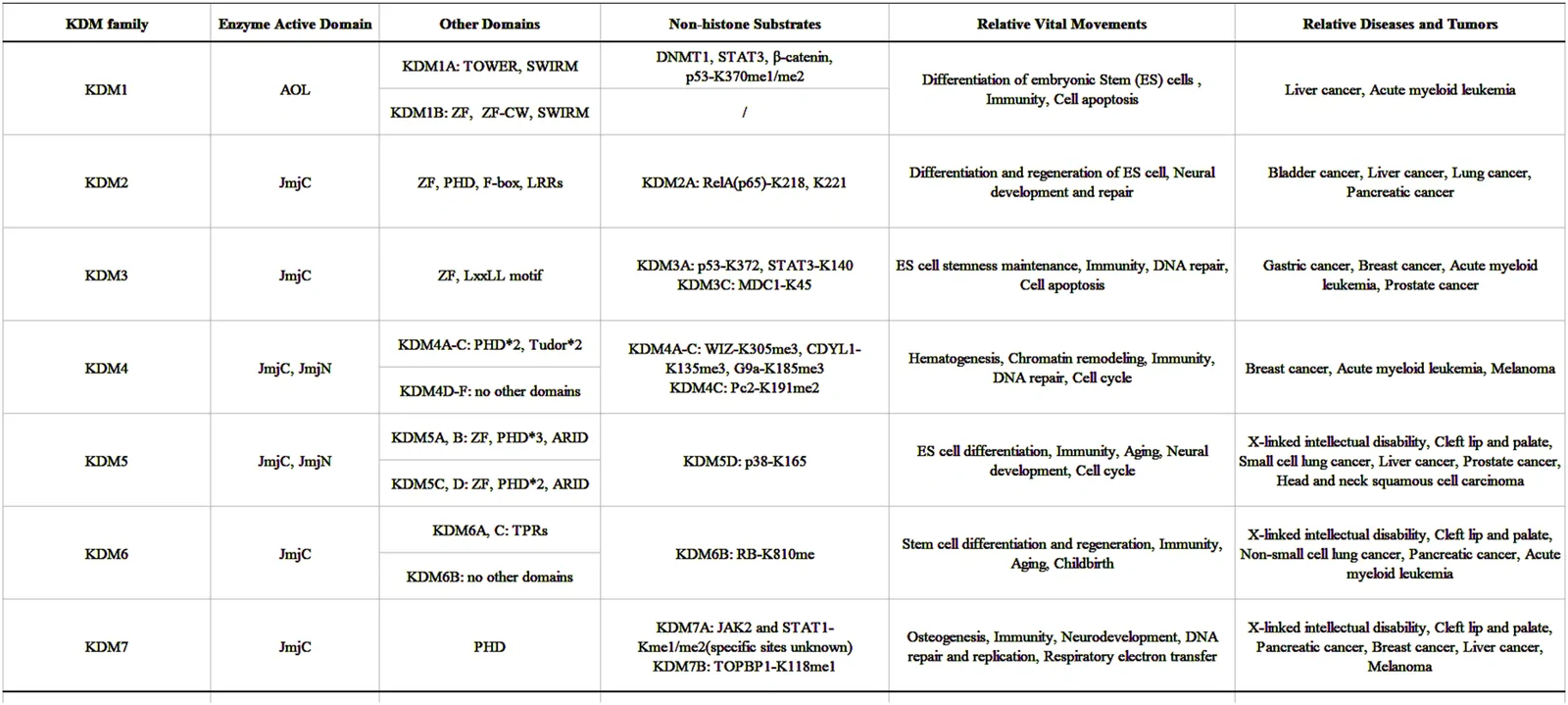
The domains, non-histone substrates and functions of KDMs family in this article.

In summary, challenges and unresolved questions persist regarding histone demethylases: these difficulties extend not only to undiscovered demethylases but also to the diverse functions and mechanisms of already identified ones. Questions remain regarding how KDM4D specifically targets and recognizes H3K9me2/me3; why X chromosome-localized KDM5C, KDM6A, and KDM7B escape X-inactivation; and why KDM5C and KDM6A have Y chromosome homologs (KDM5D and KDM6C) while KDM7B does not, among other unresolved issues. However, we believe these issues will be more reasonably explained through advancements in research methodologies and deeper investigations into these enzymes in the future.
